# Consolidated guidance for behavioral intervention pilot and feasibility studies

**DOI:** 10.1186/s40814-024-01485-5

**Published:** 2024-04-06

**Authors:** Christopher D. Pfledderer, Lauren von Klinggraeff, Sarah Burkart, Alexsandra da Silva Bandeira, David R. Lubans, Russell Jago, Anthony D. Okely, Esther M. F. van Sluijs, John P. A. Ioannidis, James F. Thrasher, Xiaoming Li, Michael W. Beets

**Affiliations:** 1 Department of Health Promotion and Behavioral Sciences, The University of Texas Health Science Center at Houston, School of Public Health in Austin, Austin, TX 78701 USA; 2 Michael and Susan Dell Center for Healthy Living, The University of Texas Health Science Center at Houston, School of Public Health in Austin, Austin, TX 78701 USA; 3 Arnold School of Public Health, University of South Carolina, Columbia, SC 29205 USA; 4 College of Human and Social Futures, The University of Newcastle Australia, Callaghan, NSW 2308 Australia; 5 Bristol Medical School, Population Health Sciences, University of Bristol, Bristol, BS8 1QU UK; 6 Faculty of Arts, Social Sciences and Humanities, School of Health and Society, University of Wollongong, Wollongong, NSW 2522 Australia; 7 MRC Epidemiology Unit, University of Cambridge, Cambridge, CB2 0QQ UK; 8 Department of Medicine, Stanford University, Stanford, CA USA; 9 Department of Epidemiology and Population Health, Stanford University, Stanford, CA USA; 10 Department of Biomedical Data Science, Stanford University, Stanford, CA USA; 11 Department of Statistics, Stanford University, Stanford, CA USA; 12 Meta-Research Innovation Center at Stanford (METRICS), Stanford University, Stanford, CA USA

## Abstract

**Background:**

In the behavioral sciences, conducting pilot and/or feasibility studies (PFS) is a key step that provides essential information used to inform the design, conduct, and implementation of a larger-scale trial. There are more than 160 published guidelines, reporting checklists, frameworks, and recommendations related to PFS. All of these publications offer some form of guidance on PFS, but many focus on one or a few topics. This makes it difficult for researchers wanting to gain a broader understanding of all the relevant and important aspects of PFS and requires them to seek out multiple sources of information, which increases the risk of missing key considerations to incorporate into their PFS. The purpose of this study was to develop a consolidated set of considerations for the design, conduct, implementation, and reporting of PFS for interventions conducted in the behavioral sciences.

**Methods:**

To develop this consolidation, we undertook a review of the published guidance on PFS in combination with expert consensus (via a Delphi study) from the authors who wrote such guidance to inform the identified considerations. A total of 161 PFS-related guidelines, checklists, frameworks, and recommendations were identified via a review of recently published behavioral intervention PFS and backward/forward citation tracking of a well-known PFS literature (e.g., CONSORT Ext. for PFS). Authors of all 161 PFS publications were invited to complete a three-round Delphi survey, which was used to guide the creation of a consolidated list of considerations to guide the design, conduct, and reporting of PFS conducted by researchers in the behavioral sciences.

**Results:**

A total of 496 authors were invited to take part in the three-round Delphi survey (round 1, *N* = 46; round 2, *N* = 24; round 3, *N* = 22). A set of twenty considerations, broadly categorized into six themes (intervention design, study design, conduct of trial, implementation of intervention, statistical analysis, and reporting) were generated from a review of the 161 PFS-related publications as well as a synthesis of feedback from the three-round Delphi process. These 20 considerations are presented alongside a supporting narrative for each consideration as well as a crosswalk of all 161 publications aligned with each consideration for further reading.

**Conclusion:**

We leveraged expert opinion from researchers who have published PFS-related guidelines, checklists, frameworks, and recommendations on a wide range of topics and distilled this knowledge into a valuable and universal resource for researchers conducting PFS. Researchers may use these considerations alongside the previously published literature to guide decisions about all aspects of PFS, with the hope of creating and disseminating interventions with broad public health impact.

**Supplementary Information:**

The online version contains supplementary material available at 10.1186/s40814-024-01485-5.

## Key messages regarding feasibility

• There are more than 160 published guidelines, reporting checklists, frameworks, and recommendations related to PFS. All these publications offer some form of guidance on PFS, but many focus on one or a few topics, making it difficult for researchers wanting to gain a broader understanding of all the relevant and important aspects of PFS and requires them to seek out multiple sources of information, which increases the risk of missing key considerations to incorporate into their PFS.

• We present a set of consolidated considerations for behavioral intervention pilot and/or feasibility studies based on a review of the literature and a Delphi study with the authors who wrote this literature.

• We believe this consolidated set of considerations can be a “go-to” resource for any behavioral interventionist wanting to design, conduct, and report on their pilot and/or feasibility study.

## Background

In the behavioral sciences, conducting pilot and/or feasibility studies (PFS) is a key step that occurs early in the translational science continuum. PFS provide essential information to inform the design, conduct, and implementation of larger-scale trials, although not all studies follow the traditional roadmap to scale-up [1]. PFS are designed to answer questions surrounding uncertainty (feasibility) and potential impact (preliminary efficacy) and to inform gaps in knowledge about the various aspects of the intervention or conduct of the study. In turn, this information is used to make decisions regarding scale-up and future plans for a larger-scale trial.

There are more than 160 published guidelines, checklists, frameworks, and recommendations related to the design, conduct, and reporting of PFS. These publications offer some form of guidance on PFS, but many focus on a specific aspect of design, conduct, and reporting considerations. This makes it difficult for researchers who want to gain a broader understanding of all the relevant and important aspects of PFS and forces them to seek out multiple sources of information, which increases the risk of missing key considerations to incorporate into their PFS. Because of this, we believe a consolidated list of considerations, drawing on the breadth and depth of knowledge that has already been published on the topic, would have high utility for researchers and assist them in understanding important considerations and nuances when conducting a PFS.

Throughout this paper, we refer to PFS as early-stage studies designed to inform larger-scale, well-powered trials. We recognize that there are numerous labels for such studies (e.g., “proof-of-concept”, “evidentiary”, “vanguard”). We also realize that the terms “pilot” and “feasibility” evoke different meanings [2, 3] and are used interchangeably and, in some instances, simultaneously. We address this issue in this consolidation of considerations. We also recognize that not all PFS will include or need to consider all the identified considerations. In many instances, however, a single PFS is designed to cover all of the data needed to inform a larger-scale trial [4]. This includes everything from estimating recruitment/retention rates, participant satisfaction and engagement, fidelity, and a host of other feasibility indicators, as well as providing some preliminary indications of change in one or more outcomes of interest. Researchers often deliberately design a PFS to collect information across these multiple dimensions, though their decision making is often largely driven by such issues as available resources and abbreviated timelines.

The purpose of this study was to develop a consolidated set of considerations for the design, conduct, implementation, and reporting of PFS for interventions in the fields of behavioral sciences. The considerations presented herein were developed through any extensive review of the literature and a Delphi study of experts who wrote the existing literature on PFS. The consolidated set of considerations was developed for universal application across interventions in the behavioral sciences and across the study designs one may choose. We expect this consolidation will serve as a valuable resource for all behavioral science interventionists who design and conduct PFS, regardless of the intervention mechanism, target population, or study design.

## Methods

To ensure rigor and methodological quality throughout the consolidation of previously published guidelines, checklists, frameworks, and recommendations, we relied on guidance from Moher et al., [5, 6] which details the main steps in the development of evidence-based consensus in health fields. These steps included developing a strong rationale for the consolidation, necessary preparatory work conducted by the study team, consensus activities, and development of the final consolidation. These steps are detailed below. When relevant, we also drew on similar consensus studies conducted in the behavioral sciences [2, 3, 7, 8].

### Review of previously published guidelines, checklists, frameworks, and recommendations for PFS

A scoping bibliometric review of published PFS-related guidelines, checklists, frameworks, and recommendations was conducted prior to developing the Delphi survey, which has been reported elsewhere [9]. Briefly, we identified 4143 PFS from which we then identified 90 guidelines, checklists, frameworks, and recommendations cited in that literature. We then continued searching for relevant literature via backward citation tracking of known publications, including the CONSORT Extension for Pilot and Feasibility Studies [7], Medical Research Council guidance [10], and publications such as Bowen et al. [11] and Pearson et al. [12] A total of 161 publications were identified that encompassed nine thematic domains: *adaptations*, *definitions of pilot and feasibility studies*, *design and interpretation*, *feasibility*, *implementation*, *intervention development*, *progression criteria*, *sample size*, and *scale-up*. The 161 publications guided our inclusion of the sample of respondents for the Delphi survey, which is detailed in the next section. It is worth noting that after this review, we identified an additional relevant publication published after the completion of the study, which is included in our final sample (bringing the total number of studies to 162) but was not used to inform the Delphi study.

### Participant selection and recruitment for the Delphi survey

**Fig. 1:**
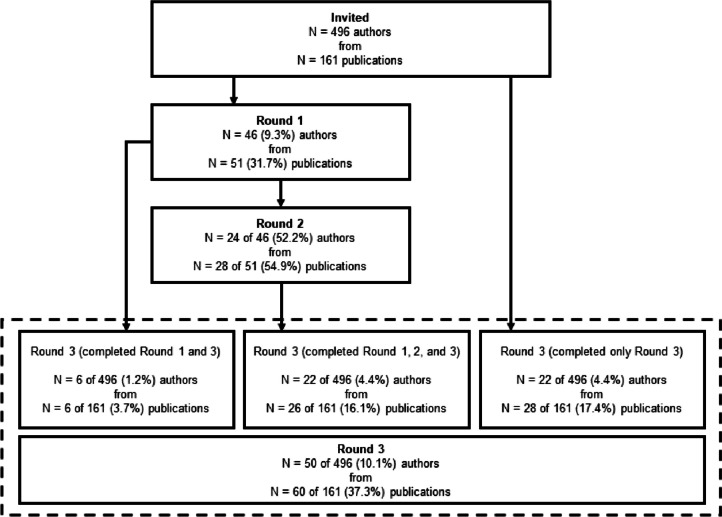
Participant flow through each round of the Delphi survey process

Lead, second, corresponding, and senior authors of the 161 published guidelines, checklists, frameworks, and recommendations for PFS were invited via email to complete a three-round Delphi study. Contact information was retrieved from published article meta-data and when not found in the published articles, emails were retrieved from another publicly available source, such as faculty pages or university websites. This resulted in 496 potential participants, who were sent an individualized invitation email via Qualtrics for round 1 of the Delphi study. For round 2, only participants who completed round 1 were invited to take part in the survey. We then sent the round 3 survey back to the original pool of 496 potential participants, regardless of whether they completed round 1. This process is summarized in Fig. 1 and took place between May 2022 and January 2023. Ethical approval was granted by the University of South Carolina’s Institutional Review Board (IRB # Pro00120890) prior to the start of the study.

### Delphi survey

Each round of the Delphi survey process was guided by established protocols [13, 14] and is detailed below.

#### Round 1—Delphi survey

In round 1 of the Delphi process, participants were asked to provide the most important considerations regarding the design, conduct, analysis, or reporting of behavioral pilot and/or feasibility intervention studies in separate free-text fields via Qualtrics. Before beginning the survey, participants were provided with operational definitions of both “behavioral interventions” and “preliminary studies” for context. No other prompts were provided. In round 1 of the Delphi study, we referred to PFS as “preliminary” studies, but after receiving comments about the use of this term, this was changed to “pilot and/or feasibility” studies in round 2. Survey distribution for round 1 took place in May and June 2022.

#### Preparation for round 2

Participants’ responses from round 1 were exported from Qualtrics to a.csv file in Microsoft Excel, collated into individual Microsoft Word documents for each participant, converted to PDFs, and imported into NVivo for thematic coding. Prior to coding responses in NVivo, we simplified and revised our original nine thematic domains from the scoping bibliometric review into six overarching themes: *intervention design*, *study design*, *conduct of trial*, *implementation of intervention*, *statistical analysis*, and *reporting*. This revision was conducted after an initial review of responses from round 1 of the Delphi survey in an effort to simplify themes and to allow for maximum parsimony across expert perspectives. Specifically, we identified overlap in several of the original nine themes and made a decision to include them as subthemes in the revision to six overarching themes. The titles of the original nine thematic domains were largely retained and can be found embedded as subthemes in the six revised overarching themes. A two-step thematic coding process followed. First, individual participant responses were coded into a corresponding theme based on the content of their response. This was completed by two members of the research team (CDP and MWB). Disagreements were brought to the larger research team (LV, SB, and AB) during weekly meetings and were resolved at that time. Once participant responses were coded into one of the six overarching themes, our research team coded responses into one of 20 subthemes based on qualitative analysis of participants’ responses by theme. These 20 subthemes served as the coding framework for the second step of the thematic coding process, and responses were coded as such by two members of the research team (CDP and MWB).

#### Round 2—Delphi survey

In round 2 of the Delphi study, participants were re-oriented to the study with a brief narrative and were presented with the six overarching themes and 20 subthemes generated via qualitative analysis of the results from round 1. To give participants context, we provided select, representative quotes for each subtheme from round 1 of the survey. After being presented with the theme, subtheme, and select quotes, participants were asked to provide a recommendation for each subtheme for inclusion in a consolidated framework for behavioral intervention PFS. Participants were also given the chance to indicate if they felt a subtheme should not be included in a consolidated framework. The survey was organized such that each theme (along with the corresponding subthemes) was presented as a randomized block, meaning individual participants were presented with a unique order of themes and asked to provide their considerations. Block randomization of themes was performed to prevent the possibility of homogenous burnout across participants as they reached the last theme of the survey. The last question of the survey was a free-text field in which participants could indicate if there were any additional considerations that were not mentioned in the survey that should be added to a consolidated framework for pilot and/or feasibility behavioral intervention studies. Survey distribution for round 2 took place in September and October 2022.

#### Preparation for round 3

Participant responses from round 2 were exported from Qualtrics to a.csv file in Microsoft Excel and collated into individual Microsoft Word documents for each of the 20 subthemes. A collection of considerations for each subtheme was written based on participant responses from rounds 1 and 2 and from information provided throughout the previously identified 161 pilot and/or feasibility-related guidelines, checklists, frameworks, and recommendations. Weekly research group meetings were used to further refine the considerations.

#### Round 3—Delphi survey

In the final round of the Delphi study, participants were first asked to provide basic demographic information including age, sex, race/ethnicity, and the year in which they received their terminal degree. Demographic information was not collected from participants in round 1 or 2 of the Delphi survey to limit participant burden in the initial rounds of the survey. We then provided participants with an outline of the six themes and 20 subthemes that emerged from rounds 1 and 2 of the study, a description of the final recommendation for the study, and instructions for the final survey. For each of the 20 subthemes, participants were given an operational definition of the subtheme and a list of considerations, which were generated based on the comments from rounds 1 and 2. They were then asked to rate their level of agreement with the considerations (0–10 Likert scale from Strongly Disagree to Strongly Agree). An optional free-text field was provided for additional information about what we should add to/change about the considerations. Participants were presented with each subtheme in block-randomized order just as in round 2. Survey distribution for round 3 took place in December 2022 and January 2023.

### Final consolidation of considerations

The final set of considerations was written in a similar manner to round 2. Responses were collated into separate working documents for each of the 20 subthemes, which also included the list of previously written considerations drafted for round 2. The previously written considerations were altered based on participant feedback from round 3 and from further supporting information from the 161 pilot and/or feasibility-related guidelines, checklists, frameworks, and recommendations. Primary changes to the considerations were made by two members of the research team (CDP and MWB) and further refined by members of our larger research team (LV, SB, and AB).

### Analysis of quantitative data

There were two forms of quantitative data gathered from participants during round 3 of the Delphi survey process. The first was demographic information, which was summarized descriptively as means, standard deviations, and ranges where appropriate. The second were the participant’s Likert-scale ratings of each set of considerations for each of the 20 subthemes. These data were summarized visually with boxplots and descriptively with means, standard deviations, medians, ranges, and interquartile ranges. All quantitative analysis was performed in STATA v17.0 statistical software package (College Station, TX, USA).

## Results

### Participant characteristics and survey completion

A total of 46 of the 496 (9.3%) invited authors representing 51 of the 161 (31.7%) identified publications completed round 1 of the Delphi study. In round 1, where respondents were asked to provide up to 20 considerations regarding the design, conduct, analysis, or reporting of behavioral pilot and/or feasibility intervention studies, participants gave a mean of 8 ± 4 (range = 1–20, median = 7, IQR = 5–10) considerations. Of the 46 participants who completed round 1, 24 (52.2%) completed round 2. A total of 50 (10.1%) of the original pool of 496 participants representing 60 (37.3%) publications completed round 3. For the 161 publications that were represented by authors in the Delphi study, the median year of publication was 2015 (range = 1998–2022, IQR = 2013–2018). Comparatively, across all possible 161 identified publications, the median year of publication was 2013 (range = 1989–2022, IQR = 2009–2017). A visual summary of participant flow through each of the three rounds of the Delphi survey process is provided in Fig. 1. Demographic information for participants who completed round 3 is presented in Table 1.

**Table 1:** Demographic information for respondents (*N* = 50) of round 3

Characteristic	Mean or *N*	SD or %	Range
Age (years)	53.8	13.5	31–85
Sex			
Female	24	48.0%	-
Male	25	50.0%	-
Not reported	1	2.0%	-
Race			
African Canadian	1	2.0%	-
Asian	1	2.0%	-
Mediterranean	1	2.0%	-
White	46	92.0%	-
Not reported	1	2.0%	-
Hispanic			
No	48	96.0%	-
Yes	1	2.0%	-
Not reported	1	2.0%	-
Years since terminal degree	23.2	13.5	5–60

### Likert ratings of the considerations

Likert scale ratings (0–10 scale) of each of the considerations for the 20 subthemes were provided by 50 out of 50 (100%) participants during round 3 of the Delphi survey. These are summarized in Table 2. Average ratings for considerations across all 20 subthemes ranged from 7.6 to 8.8, with medians ranging from 8 to 10.

**Table 2:** Summary of round 3 Likert scale ratings of considerations

Theme	Subtheme	Mean	SD	Median	Range	IQR
Intervention design	Adaptations and tailoring	8.28	1.88	9.0	0–10	8–9
	Site selection and context	8.32	2.00	9.0	0–10	8–10
	Stakeholder engagement and co-production	7.82	2.69	9.0	0–10	6–10
	Theory usage	7.56	2.42	8.5	0–10	5–9
	Well-defined problem and aims	8.46	2.00	9.0	0–10	8–10
Study design	Iteration and intervention refinement	8.52	1.58	9.0	3–10	8–10
	Progression criteria	7.86	2.60	9.0	0–10	7–10
	Randomization and control groups	8.38	2.27	9.0	2–10	8–10
	Scale-up	8.14	2.22	9.0	0–10	7–10
Conduct of trial	Measurement and data collection	8.76	1.46	9.0	4–10	8–10
	Recruitment	8.70	2.04	9.0	0–10	8–10
	Retention	8.82	1.75	10.0	2–10	8–10
Implementation of intervention	Acceptability	8.62	1.48	9.0	5–10	8–10
	Fidelity	8.72	1.80	9.0	2–10	9–10
	Cost and resources	8.10	1.85	8.0	3–10	7–10
Statistical analysis	Sample size	7.88	2.37	9.0	0–10	7–10
	Preliminary impact	7.60	2.56	8.0	0–10	7–10
Reporting	Pre-registration and protocol publishing	8.78	1.75	10.0	3–10	8–10
	Study labeling	8.50	2.56	10.0	3–10	9–10
	Framework and guideline usage	8.58	1.98	9.0	2–10	8–10

### Consolidated considerations for PFS

For each subtheme, we provide an operational definition of the subtheme, a consolidated list of considerations based on the review of pilot and/or feasibility literature and the three-round Delphi study, and a narrative summary of the subtheme. We also provide a crosswalk of 161 guidelines, checklists, frameworks, and recommendations, mapped on to the subthemes identified and an additional publication that was published after the Delphi process, but was relevant to include in the list [15]. The crosswalk is found in Additional file 1 and can be used to identify supporting literature for each of the subthemes and considerations we have consolidated. Of the 161 publications, 15 are reporting guidelines/checklists, 44 are guidelines/recommendations, 18 are reviews that offer recommendations, 37 are frameworks/models, and 47 are commentaries/editorials that offer recommendations or guidance for preliminary studies. For the narrative summary, wherever possible, we have identified relevant examples across widely used study designs for PFS which range from “N of 1” studies, micro-randomized trials, single and multiple group designs, and those involving traditional randomization, to highlight the universality of the consolidated considerations.

### Intervention design

#### Adaptations and tailoring

##### Definition

Adaptations and tailoring refer to any deliberate changes to the design or delivery of an intervention, with the goal of improving fit or effectiveness in a given context [16].

##### Considerations

- Where components of the intervention are adapted/tailored, details of who was involved (e.g., investigative team, key stakeholders, participants) in the decisions (see 1.3. Stakeholder Engagement and Co-Production), when the adaptations/tailoring occurred, and how and why the modification(s) were made need to be clearly reported.
- How the proposed adaptations/tailoring address the issues/challenges observed in the intervention need to be clearly reported along with justification for why these changes should result in an improved design.
- Whether the adaptations/tailoring occurred a priori or during the conduct of the study should be clearly described.
- The intervention component of PFS can be conducted in a rigorous fashion yet be flexible enough to allow for minor adaptations or tailoring (in composition, format, design, etc.) when justified and in response to emerging feasibility indicators.
- If substantial adaptations are made to the intervention, such that the adaptations may influence feasibility indicators or behavioral outcomes, re-testing of the PFS prior to progression is justifiable (see 2.1. Iteration and Intervention Refinement). Adaptations/tailoring occurring under these circumstances should refer to any a priori progression criteria specifications (see 2.2. Progression Criteria).

Often, existing evidence-based interventions are modified (i.e., adapted/tailored) for delivery to a new sample or in a new setting that is different from where the intervention was originally implemented and evaluated. In these situations, a PFS may be conducted to establish whether the modifications are appropriate in the new sample/setting [17, 18]. Adaptations are often made to increase relevance and participant engagement, with the assumption the adaptations would lead to better outcomes in the target populations and settings of eventual interest [19, 20].

Adaptations can consist of changes to intervention materials to make them culturally relevant to the target population (race/ethnicity, country/setting, norms/values) [19, 21]. Adaptations may also include changes to the intervention itself, such as how it is delivered (e.g., combining sessions, online vs. face-to-face), delivery location, who it is delivered by, or the length of the sessions/intervention [22, 23]. Adaptations may occur at any point in the design, implementation, and evaluation/interpretation of a PFS. These include a priori adaptations of existing interventions, those that occur as a result of the evaluation of an intervention, or adaptations made on an ongoing basis throughout a PFS [19, 21, 24–33].

Where adaptations/tailoring occur, reasons for the adaptations and who participated in the decision-making process should be reported. Often, the adaptation process includes coproduction/codesign methods that can involve focus groups, feedback sessions, and key patient, participant, and public involvement [17] to justify and inform the relevancy of the adaptations [19, 34–36] (see 1.3. Stakeholder Engagement and Co-Production). If coproduction/codesign methods are used, these should be clearly reported.

#### Site selection and context

##### Definition

Site selection refers to the location in which a PFS will be conducted. Context refers to the factors that form the setting of the intervention, including location, culture, environment, and situation [12, 37].

##### Considerations

- Whenever feasible, researchers should choose sites for PFS that are representative of those anticipated in the future larger-scale trial.
- Purposeful selection of sites can be used to ensure an intervention is tested in an appropriate range of contexts.
- A rationale for the sites selected should be clearly stated along with how the sites and context reflect what is anticipated in the future larger-scale trial.
- Key characteristics of the sites and context should be reported.
- The context of intervention delivery and any information that suggests contextual elements may impact the feasibility or future efficacy of the intervention should be clearly reported.
- Where context is known or hypothesized to influence the implementation and/or feasibility of an intervention, including more than one site may be necessary.

Setting and contextual characteristics are known factors that can influence intervention outcomes. For PFS testing interventions that rely on a setting as part of the delivery process or are embedded naturally within existing settings, site selection and context become key factors to understand at the early stages of the design and evaluation of an intervention. Setting and context may represent static (e.g., hospital serving low-resource area) or dynamic (e.g., weather, day-to-day variability) characteristics [38]. Reasons why sites are selected in a PFS can include a range of pragmatic considerations. These include the need for representation of a diverse range of characteristics (e.g., geography, populations served), facilities/infrastructure required for the project (e.g., cell phone connectivity, low-resource settings), and proximity to the investigative team [39–45]. These decisions may also be based on the ability to refer sufficient numbers of participants at a given site [43, 46, 47]. Descriptions of the context and setting and how these might influence intervention outcomes should be clearly reported [38, 48, 49].

In some PFS, understanding setting complexity and how an intervention fits within a broader system may be the primary research questions that need to be answered prior to conducting a larger-scale trial. Studies investigating setting or context are useful for the identification of whether an intervention is appropriate or feasible to deliver for a given setting [50–53]. This allows for understanding uncertainties about the setting and how differences across settings may influence implementation [54–57]. In some situations, where an existing intervention is adapted to be delivered in a different setting, understanding how the intervention interacts with the new context becomes a key feasibility outcome to evaluate.

#### Stakeholder engagement and co-production

##### Definition

Stakeholder engagement and co-production refers to the use of partnerships with individuals, communities, and service providers to aid in the development and implementation of an intervention [58].

##### Considerations

- PFS should be, whenever possible, co-designed/co-created or informed by key stakeholder (e.g., community and professional) perspectives throughout all stages of design and implementation.
- Whenever possible, pro-equity approaches that ensure the unique considerations and perspectives around an intervention’s acceptability, safety, etc., and participation in and ownership of research from minority and vulnerable populations, should be used.
- The processes by which the PFS was co-designed, including who was consulted, why, when they were consulted, and how their input was obtained, should be clearly described.

Stakeholder engagement and co-production methods are commonly used in PFS to ensure the relevance of a number of intervention-related facets. These include the relevance of intervention materials, how an intervention is delivered, whether the content is appropriate, and if any important components are missing [59–61]. Employing stakeholder engagement and co-production methods can be useful to ensure ownership of the developed intervention by recipients and end-users [62]. Where these methods are employed, it is important to report who is involved in co-production (participants, interventionists, members of the public, other key stakeholders) and a rationale for their involvement in the process [63–65]. The process of engaging stakeholders in co-production can take many forms, including “think aloud”—commonly used for useability testing, questionnaires, and/or interviews [66–70]. What participants did during the co-production process, such as reviewing qualitative interviews or initial testing of intervention materials, should be reported. Details of how participants were engaged in the co-production (e.g., time dedicated, number of rounds of review/workshops, the total number of individuals involved) should also be included [71, 72]. In some instances, it may be appropriate to describe details of the training required to facilitate a co-production process [61].

#### Theory usage

##### Definition

Theory usage refers to the utilization of any conceptual or theoretical model to inform aspects of the PFS that are mechanisms of change [8].

##### Considerations

- Researchers, where relevant, should include details about one or more behavior change theories (e.g., intervention activities, mechanisms) which informed aspects of the PFS, including whether components of the intervention are theoretically or practically informed.

The theoretical foundation of an intervention should be clearly stated. The components of an intervention may directly map on to one or more theories of change. These could be specific theories, mechanisms, or conceptual frameworks informed by practice. Theories of change should refer to intervention resources, activities, mechanisms, and intermediate and final outcomes. This information can be presented in the form of a logic model of change or conceptual frameworks depicting the theory of change or program theory [50, 73–80]. Details of the theory of change and how this informed intervention development can be presented alongside pilot and/or feasibility outcomes, but could also be published separately, such as in a protocol overview [81, 82].

#### Well-defined problem and aims

##### Definition

Well-defined problems and aims refers to the focused research questions/objectives used to guide the design, conduct, and analyses of PFS [8].

##### Considerations

- PFS should be guided by clear and focused research questions related primarily to the feasibility of the intervention and prospects of subsequent scale-up to a larger-scale trial. These well-formulated research questions should be answered by an appropriate and transparent methodology that uses both quantitative and qualitative data.
- Where appropriate, the PFS proposal and report should define a clinically important public health problem for which researchers are designing, refining, or adapting an intervention.

PFS are designed primarily to answer key aspects regarding the feasibility of an intervention. These include addressing uncertainties about the intervention and the implications of the findings for larger-scale trials [83]. Questions of uncertainty are the basis for well-defined problems and aims of PFS. These can include understanding researchers’ access to the population of interest (recruitment); acceptability of randomization (for certain study designs); developing, refining, and finalizing intervention protocols; acceptability of the intervention for the target population; intervention deliverers and other key personnel; and other feasibility-related outcomes including fidelity, cost, equity, and cultural appropriateness [70, 84–87].

In certain situations, the aims of a PFS can be more exploratory in nature. But this does not preclude the study from having a set of well-defined problems and aims. Examples may include learning about the assets, values, and/or history of the community in which an intervention could potentially be delivered and learning about the processes in which co-design and collaboration with community members could naturally take place prior to delivering an intervention.

### Study design

#### Iteration and intervention refinement

##### Definition

Iteration and intervention refinement refers to the re-testing of an intervention in PFS to further refine intervention components before scaling to a larger trial [88].

##### Considerations

- If the conclusion of the PFS is to make significant adjustments to either the study design or the intervention, then it should be acknowledged that the results do not justify proceeding further and a second PFS is necessary to establish feasibility before testing the intervention in a larger-scale, well-powered trial. Any potential changes (adaptations/tailoring) should be clearly documented along with information about how and why the changes are to be made (see 1.1. Adaptations and Tailoring).
- The decision to conduct multiple iterations of a PFS can be pragmatic or theoretical and based on factors including the perceived confidence the redesign will sufficiently address the identified problems.
- Conclusions from a PFS should include whether the intervention, in its current form, is ready for a future trial or if modifications are needed (and if so, details of them), and whether they are substantial enough to warrant another PFS.

Iterations refer to the re-testing of an intervention in another PFS [89–92]. This can be done based upon findings from a previous PFS trial where minor and/or major adjustments to the intervention, its delivery, or other aspects of the study were found. Initial evaluations of an intervention may even pre-plan for multiple iterations. The iterations create a sequence of trialing and modifying prior to any well-powered trials. At the conclusion of a PFS, investigators can make the decision, based upon progression criteria and other findings, whether additional testing of the intervention needs to ensue prior to scale-up. This decision should be left to the interventionists and co-developers and be based on the evidence collected from the PFS, available resources, and time. Decisions can be pragmatic but also important are theoretical considerations that can inform whether or why alterations to the intervention may or may not result in anticipated or unanticipated changes.

##### Progression criteria

###### Definition

Progression criteria are a set of a priori benchmarks or thresholds regarding key feasibility markers that inform decisions about whether to proceed, to proceed with changes, or not to proceed from the PFS to a future study, either a main trial or another PFS [15].

###### Considerations

- PFS should include a set of progression criteria which are used to inform decisions about whether to proceed, proceed with changes, or not to proceed to a larger-scale study.
- Progression criteria should be determined a priori and be based on either evidence from previously published/conducted research or a sound rationale provided.
- Decisions on whether to proceed should also be informed by contextual, temporal, and partnership factors that evolve over the course of the pilot and/or feasibility.
- Progression criteria should be made for feasibility metrics such as recruitment rate, retention/drop-out rate, acceptability, implementation/fidelity, and other appropriate feasibility indicators where appropriate.
- Progression decisions can also include evidence of potential impact (see 5.2. Preliminary Impact).
- Progression criteria decisions can be in the form of a “Go/No Go” system or a “Stop Light” (red/amber/green) system, indicating no progression, progression with changes, or progression with no changes.
- Deviations from the application of progression criteria may be justified if researchers are confident that a proposed solution will address the problem at a larger scale and can provide strong theoretical and/or empirical evidence to support their assertion (see 1.1. Adaptations/Tailoring).

Across all feasibility metrics, some form of progression criteria thresholds and classification systems should be pre-defined [74, 80, 93–98]. The thresholds are commonly study- and intervention-specific, and these thresholds can be designated by investigators and any co-designers. Common classification schemes include red/amber/green and go/no-go. Often, these criteria are pre-registered and/or appear in protocol documents. Progression criteria can be used to gauge whether certain aspects of the intervention and its delivery along with other aspects of the study need to be modified. This information can be used to inform decisions about whether a subsequent test of the intervention should be conducted in another PFS (see 2.1. Iteration and Intervention Refinement).

##### Randomization and control groups

###### Definition

Randomization refers to the process of using random chance to allocate units (individuals or settings/clusters) to one or more intervention conditions. Randomization can be used to separate units into distinct groups or randomization within a unit for when and what intervention(s) they may receive (order and timing). A control/comparator condition serves as the counterfactual. A control/comparator group is a group of participants (and/or settings/clusters) allocated to receive differing amounts, orders, or types of intervention(s) being tested [99–101]. A baseline period can serve as a control/comparator condition for studies employing single-arm or individual-level interventions (e.g., N-of-1) [102].

###### Considerations

- Not every PFS needs to include two or more groups or employ random allocation.
- The presence of a control/comparator group or randomization can be included if it reflects the aims and objectives of the study.
- Control groups can take numerous forms and should be reflective of the objectives of the study, the context within which the intervention is tested, and acceptability by the target population.
- When randomization is employed, methods of randomization should be clearly described to ensure reproducibility.
- If a control/comparator group is present, feasibility indicators collected on the intervention group should also be collected on the control group where appropriate (e.g., feasibility of data collection, acceptability of randomization, retention).

PFS can employ a range of designs. These include N-of-1 [103], micro-randomized trials [104], single-group [105], quasi-experimental [106], and multi-group/multi-setting designs [107]. Despite these design options, not every PFS needs to employ randomization or include more than one group. The use of randomization and multi-group design features should be based on the objectives of the PFS. Randomization in PFS can take the form of allocating groups to different interventions or varying levels of the same intervention (doses). Randomization can also take the form of within-person or group allocation of the timing and/or varying interventions participants may receive. Where multiple groups are included, “what” they receive (i.e., allocated to) should be based on the nature of the intervention and be consistent with conventions within the field of study. This can range from a purely no-treatment comparator to standard practice to alternate active interventions. Where some form of a comparator group is used, researchers should evaluate feasibility metrics to understand such things as the ability to retain those not receiving the intervention and acceptability of randomization. Incorporating either randomization or multiple groups can increase the scientific rigor of the PFS but is not necessary to evaluate most feasibility metrics of an intervention.

##### Scale-up

###### Definition

Scale-up refers to the process of delivering and evaluating an intervention in progressively larger studies, beginning with testing an intervention within one or more PFS and moving towards larger studies of the same, or similar, interventions. It is a “deliberate effort to increase the impact of successfully tested health intervention so as to benefit more people and foster policy and program development on a lasting basis” [108, 109].

###### Considerations

- PFS should be designed with the intent for future testing of an intervention in large-scale trials and beyond.
- Researchers should consider plans for later-phase research on the intervention and explain how information gathered from the PFS will be used to answer key questions surrounding the uncertainty of the intervention or the design or conduct of a progressively larger future study.
- Issues regarding the adoption, implementation, and maintenance of the intervention over progressively larger studies can be considered at both the design and conduct phases of the PFS.
- Efforts should be made to ensure key features of the PFS be similar to those in the future large-scale trial. These include the amount of support to implement the intervention, characteristics of who delivers the intervention, the target population, the duration under which the intervention is tested, and the measures employed.
- Where differences are anticipated between pilot and/or feasibility testing and the larger-scale trial, a description of these differences should be provided along with a clear justification of how the changes may or may not impact the intervention.

PFS should be designed and conducted with the idea the information collected will be used to inform the testing of an intervention in progressively larger sample sizes and/or settings [85, 110–116]. This implies researchers who conduct PFS intend to continue to refine and optimize an intervention for maximal impact along a translational science continuum [117–119]. With this in mind, understanding early on how an intervention could be delivered to progressively larger numbers of individuals and/or settings should be incorporated into the early stages of the design and conduct of PFS. Considerations for scaling can include characteristics of those who deliver an intervention, the resources required to train and deliver an intervention, and to whom an intervention is delivered. How these aspects can change as one progresses from commonly smaller-sized PFS to evaluating an intervention for broader population-level impact should inform what transpires in a PFS. Researchers should, therefore, consider whether what they can accomplish on a smaller scale can similarly be accomplished on a larger scale [120, 121].

### Conduct of trial

Measurement and data collection

#### Definition

Measurement and data collection refer to any tools, devices, instruments, personnel, and time required to assess feasibility or outcomes related to an intervention.

#### Considerations

- PFS can assess the feasibility and appropriateness of measurement and data collection procedures including the following:
  - How or if the data can be collected
  - The acceptability of the measurements and data collection procedures (e.g., burden)
  - If the measures are valid for the population/outcomes in question

- Where applicable, measurements and data collection procedures should closely resemble those anticipated for the well-powered trial.
- The reporting of measurement and data collection procedures should be sufficiently detailed to permit standardized data collection, including information about why the measurements were selected and how they were administered, scored, and interpreted.
- Information about the feasibility and appropriateness of measurement and data collection procedures can consist of both quantitative and qualitative data sources.

The process of collecting outcome data in a PFS serves to demonstrate the feasibility of data collection methods—whether explicitly stated or not [122]. However, some PFS may be designed to answer whether outcome measures proposed for the larger-scale trial can be collected. This can include the ability to collect data using more invasive/burdensome methods (e.g., urine/hair samples, blood draws) [123, 124]. Additional metrics associated with the feasibility of measurement and data collection may include determining rates of missing data, participant response rates, and any time/resource costs associated with data collection [125–127]. This information can be used to reduce participant burden and costs associated with data collection as well as refine protocols in the larger-scale trial [128–136].

#### Recruitment

##### Definition

Recruitment refers to the procedures used to identify and select potential participants (individuals and/or settings/clusters) and enroll them into a PFS. The recruitment rate is the proportion of eligible participants or settings/clusters who are enrolled at the baseline of an intervention trial compared to the invited/eligible target population [137].

##### Considerations

- Recruitment procedures should be clearly described, with any strategies designed to maximize recruitment fully detailed.
- Information should include details of procedures used to recruit at the individual and setting/cluster levels, where appropriate.
- Recruitment information should include the following, where appropriate:
  - Proportion of eligible units (e.g., individuals, settings) recruited
  - The start and end dates of the recruitment periods
  - Number of participants recruited per setting/cluster, overall, and number of settings/clusters
  - Number of potential participants screened, eligible, consented, and enrolled in the study
  - Reasons for non-recruitment/non-consent
  - Acceptability of recruitment strategies
- Details should be provided about the recruitment strategies used, measures of their success, what worked, and what may need to be altered for future studies.

Participant recruitment is a key marker of intervention feasibility. Identifying optimal recruitment strategies in a PFS plays a critical role in determining whether the specified sample size can be achieved in the well-powered trial. Recruitment strategies may include opt-out methods (passive consent), telephone reminders, open designs (participants know what arm of the trial they are in), referrals, modalities of communication with potential participants (e.g., phone calls, emailing, text, mailings), convenient study location, and partnering with community members/settings [138–141]. The specific recruitment strategies used can influence the demographic makeup of participants. Different recruitment strategies can also yield varying amounts of eligible participants. In addition, each recruitment strategy has an associated cost. It may also be important to identify reasons why participants refused to participate in the study and record these reasons quantitatively and/or qualitatively. This information should be collected at the individual and/or setting level where appropriate. These can be important to establish during a PFS to optimize recruitment procedures in the larger-scale trial, especially in situations where there are uncertainties around recruiting the target population. At times, it may even be appropriate to formally test recruitment strategies, particularly when there is uncertainty about the best approach. For example, by embedding a “Study Within A Trial” (SWAT), researchers may gain answers to uncertainties around methodological decisions regarding a number of feasibility outcomes, including recruitment [142, 143].

#### Retention

##### Definition

Retention (attrition/drop-out) is the proportion of enrolled participants who are present throughout the full length of the intervention [137].

##### Considerations

- Researchers conducting PFS should ensure retention rates are measured.
- Where possible, assessments can be made to identify differences in retention across groups or intervention conditions.
- Reasons why individuals leave a study can be collected and analyzed to investigate whether particular factors are associated with retention.
- Procedures should clearly describe strategies used to assist with retaining participants during the delivery of the intervention and any post-intervention follow-up time periods, where appropriate.
- Retention-related information can include both quantitative and qualitative data sources.

Retention is a commonly assessed marker of intervention feasibility. Retaining participants throughout an intervention is important to ensure participants receive the full dose of intervention components as designed and whether selective attrition is present. Retention-related information also helps to understand issues regarding missing data and low statistical power in future studies. Ultimately, retention is a marker of intervention viability. In other words, if participants do not want to receive an intervention it is unlikely to be impactful.

For a given intervention, a clear definition of retention should be reported. This can include participants staying for the duration of study-related procedures/measures (e.g., data collection), completing intervention components, and/or attendance at intervention sessions [22, 92, 128, 144]. Depending on the nature of the intervention and the outcomes targeted, PFS may be designed specifically to address issues regarding retention in samples that have been historically challenging to engage/retain in interventions [145, 146].

Retention strategies, such as flexible scheduling, reminders, compensation, consistency in study staff (continuity of relationships), gathering multiple contacts, thank you and birthday cards, and follow-up phone calls within a given period, can reduce the rate of participant drop-out [139, 147–149]. Where dropouts occur, reasons for withdrawal from the study can be collected [128, 150]. Factors influencing retention, both positively and negatively, including participant motivation/aspirations, expectations, the perceived need for an intervention, and accessibility of intervention (location delivered), can be collected from both participants and intervention deliverers [151–155].

### Implementation of intervention

#### Acceptability

##### Definition

Acceptability is a perception/notion that an intervention or various aspects of an intervention are favorable, agreeable, palatable, enjoyable, satisfactory, valued, appropriate from the perspectives of participants or communities receiving the intervention, and/or have a wider fit within a system. It relates to how users “feel” about an intervention [156].

##### Considerations

- Researchers should clearly define what is meant by “acceptability” for a given study, at what levels (e.g., individual, deliverer, setting) it will be assessed, and by what methods (e.g., surveys, interviews). This should be based on the nature of the intervention and its constituent components, target population, setting level characteristics, and key stakeholders.
- Measures of acceptability can be pre-defined and included in both the PFS and large-scale trial stages.
- Acceptability should be captured, at minimum, from the end user (intervention participants). Acceptability can also be captured from those involved with delivering the intervention, along with anyone else involved in the implementation process.
- Acceptability, as defined for a given study, can be assessed for participants in control conditions where appropriate (e.g., acceptability of randomization to active comparator, acceptability of data collection procedures).
- Researchers can use both quantitative (e.g., surveys) and qualitative (e.g., interviews) methods to assess acceptability.

In most behavioral interventions, it is important to understand whether those receiving an intervention, those delivering an intervention, and any other key individual(s) find the intervention, either in its entirely or in relevant parts, to be “acceptable” to inform whether the intervention would be used or tolerated. Acceptability encompasses a range of aspects related to impressions of an intervention. These can be gathered anytime along the intervention development continuum. At the earliest stages of conceptualization, prior to packaging and preliminary testing of an intervention, assessments of acceptability (preferences) can include participants’ views of whether the proposed intervention could be appropriate for addressing a given outcome, whether they (the participants) would be willing to adhere to an intervention, the suitability of intervention materials, or whether they perceive the intervention to be useful. During intervention delivery, ongoing assessment of likeability, satisfaction, metrics of engagement with an intervention, and utility can be collected periodically [45, 157–159]. Once an intervention is completed, post-assessment markers of acceptability can include perceptions of the length or overall burden of the intervention, what strategies/components of an intervention were liked best, referral of the intervention to others, or whether the intervention met their (the recipients, deliverers, others) preferences/expectations. Where an intervention is delivered by individuals outside the intervention-development team, assessing their perspectives on the acceptability of an intervention may be necessary.

Assessments of acceptability can include both qualitative and quantitative measures. User-centered design [160] and “think aloud” protocols [161] can be used in the early stages of intervention conceptualization/formulization. Exit interviews, upon intervention completion, from recipients, deliverers, and other key individuals involved in the intervention, are often employed to evaluate markers of acceptability. Quantitative measures typically include items developed specifically for a given study. Alternatively, existing scales assessing acceptability can be used or modified accordingly for a given application [162–164]. Acceptability can also cover other aspects of the evaluation process of an intervention. This includes such areas as whether completing the proposed measures is feasible, acceptability of being randomized, or whether recipients were satisfied with the location where an intervention was delivered.

#### Fidelity

##### Definition

Fidelity is the degree to which an intervention is delivered as intended and the quality of that delivery [165, 166].

##### Considerations

- Researchers should clearly define what is meant by “fidelity” for a given study, at what levels (e.g., individual, deliverer, setting) it will be assessed, and by what methods (e.g., surveys, interviews).
- Measures of fidelity should be pre-defined with all intervention components listed.
- Fidelity can consist of information about how an intervention will be delivered, for whom, what the intervention consists of, and when and where (context) the intervention will be delivered.
- If strategies are used to encourage fidelity (e.g., a manualized intervention, feedback to those delivering the intervention), these should be reported.
- Factors influencing fidelity can be assessed and, where appropriate, linked to feasibility outcomes.

Fidelity is often a primary marker of implementation. Assessment of an intervention’s fidelity provides key information regarding whether an intervention, either the testing of individual components or in their entirety, can be delivered as intended. In PFS where initial evaluations of an intervention are conducted, fidelity plays an important role in identifying whether the intervention can be delivered as intended. Evaluation of fidelity implies a working understanding of the intervention and some pre-planned, a priori expected delivery [167, 168]. Measuring fidelity can be useful where adaptations (or changes) to the materials may take place (either planned or unplanned). Systematically documenting deviations from the original intervention can yield important insights into whether adaptations were beneficial or detrimental to the outcomes [169].

Fidelity can include many aspects of an intervention. These include adherence to intervention materials (what was done), quality of delivery (how it was done), and the dose of what was received [166, 170]. Assessing fidelity can take many forms. This includes the creation of study-specific fidelity checklists which capture the presence of key components that should be delivered during an intervention (e.g., key material to be delivered in session one or a multi-session intervention) and how they were delivered [134, 171]. Response ranges vary from present/absent, yes/no, to Likert-scaled items. Fidelity checklists can be completed either in real-time or reviewed later through the use of recorded video or audio of completed sessions [172–174]. Checklists can be completed by either someone external to the delivery agent via structured observations/recordings or completed by the delivery agent (e.g., self-report, logbooks) immediately following the delivery [175–177].

Qualitative interviews of delivery agents can also be conducted to gauge views regarding aspects of an intervention such as the training received to deliver, confidence in delivering, and any perceived barriers to delivering an intervention as planned [172]. Factors affecting fidelity can be collected to understand what, if anything, may influence departures from delivering an intervention as designed [22, 132, 173, 178]. Common ways to encourage fidelity are through the use of a manualized package of procedures, training materials, and ongoing review of sessions accompanied by feedback.

#### Cost and resources

##### Definition

Costs and resources refer to the investments and assets required to develop, implement, and sustain an intervention [12, 179].

##### Considerations

- PFS can include assessments of the costs and required resources of conducting an intervention.
- In PFS costs and resources mIn PFS costs and resources may include the following:ay include the following:
  - Monetary costs associated with training, supervision, and recruitment of both stakeholders and participants, incentivization, facilities, materials, and intervention component development and delivery.
  - Opportunity costs/time demands associated with completing the intervention by participants and delivering the intervention by providers.
- Researchers can collect information to determine the feasibility of measuring the costs associated with the intervention, with this information used to inform a more well-defined cost analysis/economic evaluation in a larger-scale trial.
- Researchers should keep in mind that some costs associated with the intervention will be fixed (one-time costs) and some will be recurring during the successful scale-up and sustainment of the intervention.

For some PFS, collecting the costs associated with delivering an intervention may be necessary to inform a larger-scale trial. In PFS, this is often referred to as conducting an economic evaluation, costing, or cost analysis [125, 180–183]. Studies may collect cost data to “rehearse” cost-effectiveness evaluations (economic evaluations) or evaluate the feasibility of collecting cost-related data [169, 184]. Where cost data are collected, micro-costing approaches that inventory all associated costs with an intervention are often conducted and used to generate a total cost per unit estimate, often expressed as a cost per participant. Costs can be fixed, variable, or projected future estimates, and they may vary according to the desired fidelity and rigor of the implementation of the interventions. Common resources inventoried for cost include the costs of consumables, staff time, services received, transportation, room hires, and refreshments. Costs can be separated into the costs associated with the initial design/development, set up of the intervention, training of staff to deliver, and the costs associated with intervention delivery. The inclusion of cost data is not study-design specific and spans a wide range of designs from *N*of 1 to cluster randomized studies [185–187].

### Statistical analysis

#### Sample size

##### Definition

Sample size refers to the number of participants (or groups/clusters) in a given study [188].

##### Considerations

- The sample size of a PFS should be based on the feasibility objectives of the study.
- Sample sizes do not have to be based upon a formal sample size calculation (i.e., power).
- Sample sizes should be pre-specified and justified.
- Sample size estimates should consider the representativeness of the target population or subgroup, setting, and other relevant contextual aspects that may influence how and why an intervention works.
- Sample characteristics should be clearly described and may refer to individuals and/or clusters (e.g., churches, workplaces, neighborhoods, schools).
- Where relevant, studies should clearly report factors impacting the sample size (e.g., availability of funds, time constraints).
- Investigators are encouraged to report the a priori power achieved by the sample size selected for a PFS.

It is widely recognized that most PFS are underpowered to detect clinically significant/public health important effects in outcomes. Selecting the appropriate sample size for a PFS, however, can vary across studies based on the objectives. In some instances, formal power calculations can be conducted/presented, but one should avoid the temptation of presenting a PFS as being well-powered by assuming implausibly large effects and/or event rates and using non-relevant outcomes. Sample size justification can be made based on other factors including, but not limited to, the availability of resources, the number of potential participants within a given setting, representativeness of the sample to the target population, complexities regarding the intervention, or the experiences of the investigators working with the population/setting [189–193]. Regardless of the approach taken, researchers need to ensure they have sufficient numbers (i.e., sample size) to make informed decisions based on the feasibility metrics and objectives of a PFS and acknowledge any limitations that the usually small sample size confers.

#### Preliminary impact

##### Definition

Preliminary impact is the ability of an intervention, during a PFS to produce a desired or intended result [194].

##### Considerations

- PFS need not be powered to detect statistically significant differences in outcomes, but one or more outcomes, as appropriate to the research, can be assessed.
- When outcomes are collected, changes in outcome data (e.g., estimated effect sizes) can be used to aid in decisions regarding the conduct of a subsequent larger-scale trial (e.g., sample size needed).
- In many cases, it may be necessary to demonstrate an intervention “moves” outcomes in the appropriate direction and is not causing harm. In this scenario, it is recommended statistical testing can be performed but to avoid the interpretation of p values as conclusive evidence of an intervention’s impact in a larger-scale trial.
- Interpretations of performed statistical tests should not include a justification for (or against) proceeding to a subsequent large-scale intervention or for making claims about the likely success of the study. Interpretations should help guide, but not dominate, the decision to proceed to a large-scale intervention.
- Investigators should avoid misusing language such as “statistically significant” to describe their interpretation of outcomes from a PFS.
- Where pilot and/or feasibility estimates of impact on primary, secondary, or tertiary outcomes are reported these should be pre-specified, with point estimates and a measure of variability reported for all time points.
- For studies presenting both feasibility and outcome data, outcome data should be relegated to a secondary or exploratory focus.

In some circumstances, it may be appropriate to evaluate, in a preliminary/exploratory fashion, the potential impact of an intervention on proximal or distill outcomes in a PFS. Where outcomes are assessed and reported, researchers need to understand the evidence is neither definitive nor necessarily very indicative of an intervention’s impact within a larger-scale trial. Nevertheless, the evaluation of outcomes within a PFS can provide useful, additional information to help inform decisions about whether the intervention is ready to be tested at a larger scale. When reporting outcomes, researchers should avoid using misleading language centered on the presence or lack of “statistical significance”. All reported outcome assessments should be secondary to feasibility metrics, which are the primary focus of most PFS. Further, it is suggested that journals should not require by default outcome assessments and/or formal hypothesis testing for manuscripts that report on PFS nor base publishing decisions on the outcomes of potential efficacy analyses if reported.

### Reporting

#### Pre-registration and protocol publishing

##### Definition

Pre-registration and protocol publishing refers to an a priori process of documenting planned intervention design and analyses [195].

##### Considerations

- Pre-registration and a protocol made publicly available (via peer-reviewed journal, pre-print server, or other forms of public dissemination) contributes to transparency and ensures that changes between what is planned, what is conducted, and what is ultimately reported are communicated and justified.

We acknowledge there is a certain degree of flexibility when it comes to PFS between what is proposed and what actually transpires in the execution of the study. Pre-registration of PFS needs to balance the developmental/exploratory nature of these types of studies with the need to document and adhere to general protocols that are the foundation of rigorous and transparent science. The goal of pre-registration is not to create an inflexible scope of work that cannot adapt to uncertainties encountered in the study, but to communicate changes to a protocol and to justify why those changes were made.

Pre-registration of study objectives can be appropriate and at times required based upon funding stipulations. While some PFS are not pre-registered, many can be found on existing trial registries. These include Clinical Trials [196] and other emerging pre-print servers and open-science repositories, such as Open Science Framework [197, 198]. Protocol publishing is also becoming increasingly common for PFS. Pre-registration and protocol publishing may help to provide details about a PFS as well as ensure deviations, although necessary at times, are clearly documented.

#### Study labeling

##### Definition

Study labeling refers to naming/presenting a PFS with appropriate naming conventions for the study being conducted [2, 3].

##### Considerations

- At a minimum, researchers should make sure studies are clearly labeled to indicate their preliminary nature and reflect the aims and objectives of the study in both the title and abstract with either “pilot”, “feasibility”, “proof-of-concept”, “formative”, or other relevant label(s).

PFS should be clearly labeled to identify and separate them within the intervention development and evaluation literature. One of the benefits of clearly labeling PFS is the ease of identification of these types of studies to understand the evolution of behavioral interventions. Because PFS are often smaller in scale, clear identification also helps to distinguish these types of studies from studies that are small in scale and lack an emphasis on intervention development, refinement, and scaling.

A number of different taxonomies have been proposed to label these types of studies. However, we recognize researchers can and do use terms referring to preliminary studies interchangeably or utilize a combination of them to describe a single study [79, 136, 167, 199–210]. In the absence of a universal consensus of terms, it is recommended investigators clearly label their PFS with one or more widely used terms that identify the preliminary nature of the study. These terms could include “pilot”, “feasibility”, “proof-of-concept”, “preliminary”, “evidentiary”, “vanguard”, and/or “exploratory”. Thus, investigators should identify the most appropriate term(s) that describe the objective of their study. This should consider the stage and number of tests/evaluations of an intervention.

#### Framework and guideline usage

##### Definition

The utilization of published frameworks/guidelines to guide the development, implementation, and reporting of PFS.

##### Considerations

- Where possible, researchers should choose an appropriate framework to structure PFS and use it to guide the design, conduct, analysis, and reporting of said study.
- Findings from PFS should be disseminated in a way that adheres to reporting guidelines to facilitate transparency and allow for replication.

There are many existing guidelines, checklists, frameworks, and recommendations that can be useful for the design, conduct, implementation, analysis, and reporting of PFS [9, 211]. The use of these publications is associated with higher study quality and reporting [9]. Guidelines include those developed specifically for PFS and also include those designed outside of the preliminary study context but are highly relevant to many aspects of PFS. Investigators should be familiar with existing guidance and utilize it appropriately, based on the specific objectives of their PFS.

## Discussion

PFS play a pivotal role in the development, refinement, implementation, and sustainability of successful behavioral interventions. This is evidenced by their emphasis on funding agencies [4, 212–216] and depiction within translational science frameworks [117, 118, 217, 218]. We identified 161 publications offering some form of guidelines, checklists, frameworks, or recommendations for PFS. Through a Delphi study utilizing expert perspectives, we developed a comprehensive set of considerations which span the continuum of development, conduct, implementation, evaluation, and reporting of behavioral intervention PFS. We believe this will serve as a valuable resource for researchers in the behavioral sciences.

### Continued challenges with PFS

While this consolidation of considerations for PFS was developed for broad applicability, there were strong opposing views among the Delphi study participants on some of the considerations that represent continued challenges with PFS. The most striking opposing opinions were observed within the “statistical analysis” theme and were present in both the “sample size” and “preliminary impact” considerations. For example, several respondents in the Delphi study believed sample size estimates for a larger-scale trial can be informed by the estimated intervention effect sizes generated from a PFS, and formal hypothesis testing can be performed and associated *p* values interpreted in a preliminary study. Other respondents expressed strong opinions that the sample of a PFS need not be representative of the target population. Conversely, the vast majority of respondents agreed that sample size justifications should be based on the feasibility objectives of a given PFS and argued against hypothesis testing (i.e., formal statistical testing and interpretation of *p* values) during the early phases of intervention development. There have been arguments made for reporting confidence intervals instead of *p*values for any non-feasibility-related outcomes assessed during PFS [219–222]. However, respondents of our Delphi study were quick to point out there is little practical difference between the use of *p* values or confidence intervals, especially if the PFS is underpowered from the start.

Opposing views were identified throughout the Delphi process for other considerations as well, including “study labeling” and “pre-registration and protocol publishing”. For study labeling, some respondents appreciated the distinction between “pilot”, “feasibility”, and other “preliminary study” terminology, while others worried that these distinctions were not well known and may cause undue confusion. Many participants of the Delphi study indicated they would rather there be no distinction, voicing concerns that adopting rigid taxonomies would create research silos and hinder cross-purpose innovation. Ultimately, we chose not to take a definitive stance on this issue, but rather make researchers aware they should be labeling PFS in some way to aid in the identification of these types of studies. On the topic of pre-registration and protocol publishing, some Delphi respondents argued that pre-registration and protocol publishing for PFS was asking too much and that this type of work should be reserved only for larger-scale trials. Others fully supported the idea of pre-registration and protocol publishing for PFS, arguing it aids in transparency and reproducibility. Again, these are decisions ultimately left up to the researchers conducting PFS, but it is likely that registration will be increasingly requested and enforced (e.g., by funders). The lack of registration of all PFS means that one cannot understand the totality of the efforts that are made in that space for developing and assessing the feasibility of an intervention.

It is important to understand that what may be viewed as common and accepted practice may not be widely held everywhere and the reasons for this vary according to country, funder, and disciplinary norms. It may be that differing opinions stem from differences between what commonly accepted/promoted translational science frameworks espouse and the realities of conducting PFS, which are often conducted with limited resources and abbreviated timelines. In addition, there may be different levels of expectations about what is proposed in these frameworks and the expectations of funding agencies and grant reviewers [223]. Such disagreements can prove problematic for behavioral scientists when seeking funding or wanting to publish findings from their PFS. Reconciliation on these topics is unlikely, and perhaps unnecessary, yet it is important to acknowledge what can and cannot be accomplished by a PFS. We believe appropriately tending to these issues throughout all phases of design, conduct, interpretation, and reporting should help preemptively dissuade critiques that could stymie the progress of intervention development and implementation.

### Progress for PFS

While disagreements were noted for a few considerations, most respondents agreed on the content of most topics. For example, participants of the Delphi study agreed that *feasibility* outcomes, including recruitment, retention, acceptability, and fidelity should take priority over preliminary impact and should be used and presented as the primary outcomes of PFS. This also aligns with developing well-designed problems and aims of PFS, most of which should answer questions regarding uncertainties (feasibility) of an intervention. Respondents also agreed progression criteria are useful when developing and deploying PFS, although some recommended caution on the use of progression criteria that are too rigid when making decisions about scaling up PFS to the next stage. Finally, and perhaps most salient, participants agreed on the importance of PFS as a critical step in successful large-scale intervention development and implementation. However, one cannot exclude the presence of selection bias in favor of the importance of PFS among authors who have authored guidelines on them and even more so among authors who responded to our surveys.

### Use of the considerations

We believe the considerations in this paper span the continuum of PFS, from development to reporting, and will be useful for researchers planning to conduct their very first PFS to well-seasoned interventionists. We envision these consolidated considerations being used in practice and as an educational tool for trainees. On a broader scale, we are hopeful this consolidation may improve PFS in the future, reducing research waste and leading to the development of high-quality, scalable behavioral interventions with maximal reach and public health impact. In addition to the considerations themselves, we provide a crosswalk of all published guidelines, checklists, frameworks, and recommendations related to PFS in Additional file 2 in an effort to amplify the voices of experts in this field. Researchers reading this study and those who want to know more about a particular consideration are encouraged to utilize the crosswalk located in Additional file 1 to identify further reading, which may provide more specific guidance on a particular topic. While not the focus of this consolidation, we also believe many of the considerations are cross-cutting with large-scale implementation and dissemination research. Researchers doing this type of work may look to certain considerations to guide aspects of their larger-scale study as well.

### Strengths and limitations

These consolidated considerations have several strengths. First, they were created based on information gathered from 161 published guidelines, checklists, frameworks, and recommendations on the topic of PFS. We relied on authors from these very same 161 publications to voice their opinions about the most important PFS-related topics via a three-round Delphi study. The total sample of participants across three rounds of the Delphi process represented over 35% of the 161 publications. Participants had, on average, 23 years of experience since their terminal degree, representing a collective 1150 years of experience across respondents. Moreover, we supplement this consolidation with a review of those 161 guidelines, checklists, frameworks, and recommendations, creating one of the largest collective sources of information on PFS published to date. This study is not without limitations. While we had a moderate representation of Delphi participants across publications, we were only able to recruit 10% (50 out of 496 identified authors) of our target population for the Delphi process. Further, while there was an equal distribution of males and females, the sample was largely White. Other than age and years of terminal degree, we did not collect other demographic information on the Delphi participants, although the median year of publication for the publications represented in our sample was slightly more recent (2015) than the total sample of possible publications (2013) from which authors were sampled. For the considerations themselves, there is still no true consensus on many of the topics presented. Differences of opinion were observed throughout the Delphi process and can be found across the published literature. Despite this, we believe the consolidated considerations could be a valuable resource for behavioral interventionists conducting PFS on a broad range of public health topics.

## Conclusion

This is one of the first studies to attempt to garner consensus on a broad range of considerations regarding PFS for the behavioral sciences. We leveraged expert opinion from researchers who have published PFS-related guidelines, checklists, frameworks, and recommendations on a wide range of topics and distilled this knowledge into a valuable and universal resource for researchers conducting PFS. We identified 20 considerations for PFS, which fall into six categories, including *intervention design*, *study design*, *conduct of trial*, *implementation of intervention*, *statistical analysis*, and *reporting*. We also provide a list of the available publications on each of the specific considerations for further reading and use and have aligned these publications with the considerations set forth in this paper. Researchers may use these considerations alongside the previously published literature to guide decision making about all aspects of PFS, with the hope of creating and disseminating interventions with broad public health impact.

### Supplementary Information

**Additional file 1.** Literature crosswalk.

**Additional file 2.** Summary table of considerations.

## Data Availability

The datasets used and analyzed during the current study are freely available at https://osf.io/kyft7/.
