# Supplementary material for: Consolidated guidance for behavioral intervention pilot and feasibility studies
Source: Pilot Feasibility Stud. 2024 Apr 6;10:57. doi: 10.1186/s40814-024-01485-5 (PMC10998328; doi:10.1186/s40814-024-01485-5)
Supplement: Supplementary file 1 — Additional file 1. Literature crosswalk. [file 40814_2024_1485_MOESM1_ESM.docx]

| Author Year | **Intervention Design** | | | | | **Study Design** | | | | **Intervention Implementation** | | | | **Conduct of Trial** | | **Analysis** | | **Reporting** | | | **Publication Type** | | | | |
| --- | --- | --- | --- | --- | --- | --- | --- | --- | --- | --- | --- | --- | --- | --- | --- | --- | --- | --- | --- | --- | --- | --- | --- | --- | --- |
|  | Adaptations and Tailoring | Site Selection and Context | Comm. Partn. Engagement and Co-Production | Theory Usage | Well-Defined Problems and Aims | Iteration | Progression Criteria | Randomization and Control Groups | Scale-Up | Acceptability | Cost and Resources | Fidelity | Retention | Measurement and Data Collection | Recruitment | Preliminary Impact | Sample Size | Study Labeling | Framework and Guideline Usage | Pre-Registration and Protocol Publishing | Commentary/Editorial/Other | Framework/Model | Reporting Guideline/Checklist | Guidelines/Recommendations | Review |
| Abbott 2014^1^ |  |  |  |  |  |  | ⚫ | ⚫ |  |  |  | ⚫ | ⚫ |  | ⚫ |  |  | ⚫ |  |  | ⚫ |  |  |  |  |
| Albers 2018^2^ |  |  |  |  |  |  |  |  |  | ⚫ |  |  |  | ⚫ | ⚫ | ⚫ | ⚫ |  |  |  |  |  |  | ⚫ |  |
| Albrecht 2013^3^ |  | ⚫ |  | ⚫ |  |  |  | ⚫ |  |  |  | ⚫ |  |  |  |  |  |  |  | ⚫ |  |  | ⚫ |  |  |
| Algase 2009^4^ |  |  |  |  |  |  |  |  |  | ⚫ |  |  |  | ⚫ | ⚫ | ⚫ |  |  |  | ⚫ | ⚫ |  |  |  |  |
| An 2020^5^ |  |  |  |  |  |  |  |  |  |  |  |  |  |  |  |  |  |  |  |  |  |  |  |  |  |
| Arain 2010^6^ |  |  |  |  |  |  |  | ⚫ | ⚫ | ⚫ | ⚫ |  | ⚫ | ⚫ | ⚫ | ⚫ | ⚫ |  |  |  |  |  |  |  | ⚫ |
| Arnold 2009^7^ |  |  |  |  | ⚫ |  |  |  |  | ⚫ |  | ⚫ | ⚫ |  | ⚫ | ⚫ | ⚫ |  |  |  |  |  |  |  | ⚫ |
| Aschbrenner 2022^8^ |  |  |  |  | ⚫ | ⚫ | ⚫ |  |  | ⚫ | ⚫ | ⚫ | ⚫ | ⚫ | ⚫ |  |  |  |  |  |  |  |  | ⚫ |  |
| Avery 2017^9^ |  |  |  |  |  |  | ⚫ |  |  |  |  | ⚫ |  |  | ⚫ |  |  |  |  | ⚫ |  |  |  |  | ⚫ |
| Baier 2019^10^ |  |  |  |  |  |  |  |  | ⚫ |  |  |  |  |  |  |  |  |  |  |  |  |  |  | ⚫ |  |
| Barker 2016^11^ |  |  |  |  |  |  |  |  | ⚫ |  |  |  |  |  |  |  |  |  |  |  |  | ⚫ |  |  |  |
| Barrera 2006^12^ | ⚫ |  |  |  |  | ⚫ |  |  |  |  |  |  |  |  |  |  |  |  |  |  |  | ⚫ |  |  |  |
| Barrera 2013^13^ |  |  |  |  |  |  |  |  |  |  |  |  |  |  |  |  |  |  |  |  |  |  |  |  |  |
| Bartholomew 1998^14^ |  |  | ⚫ | ⚫ | ⚫ |  |  |  |  |  |  |  |  |  |  |  |  |  |  |  |  | ⚫ |  |  |  |
| Becker 2008^15^ |  |  |  |  |  |  |  |  |  |  |  |  |  |  |  |  |  | ⚫ |  |  | ⚫ |  |  |  |  |
| Beebe 2007^16^ |  |  |  |  |  |  |  |  |  | ⚫ |  | ⚫ | ⚫ |  | ⚫ |  |  |  |  |  | ⚫ |  |  |  |  |
| Bell 2018^17^ |  |  |  |  |  |  |  |  | ⚫ |  |  |  | ⚫ |  | ⚫ | ⚫ | ⚫ |  |  |  |  |  |  | ⚫ |  |
| Billingham 2013^18^ |  |  |  |  |  |  |  |  |  |  |  |  |  |  |  |  | ⚫ |  |  |  |  |  |  |  | ⚫ |
| Blatch-Jones 2018^19^ |  |  |  |  |  | ⚫ |  |  | ⚫ | ⚫ | ⚫ | ⚫ | ⚫ | ⚫ | ⚫ |  | ⚫ |  |  |  |  |  |  |  | ⚫ |
| Bond 2017^20^ |  |  |  |  |  |  |  |  |  |  |  |  |  |  |  |  |  | ⚫ | ⚫ |  | ⚫ |  |  |  |  |
| Bond 2023^21^ |  |  |  |  | ⚫ |  |  |  |  |  |  |  |  |  |  |  |  | ⚫ |  |  |  | ⚫ |  |  |  |
| Borek 2015^22^ |  | ⚫ |  | ⚫ |  |  |  | ⚫ |  |  |  | ⚫ |  | ⚫ |  |  | ⚫ |  |  |  |  |  | ⚫ |  |  |
| Bowen 2009^23^ | ⚫ | ⚫ |  |  |  |  |  |  |  | ⚫ |  | ⚫ |  |  |  | ⚫ |  |  |  |  |  |  |  | ⚫ |  |
| Braganza 2021^24^ | ⚫ | ⚫ | ⚫ |  | ⚫ |  |  |  |  |  |  |  |  |  |  |  |  |  |  |  |  | ⚫ |  |  |  |
| Bugge 2013^25^ | ⚫ | ⚫ |  |  |  |  |  | ⚫ |  | ⚫ | ⚫ | ⚫ | ⚫ | ⚫ | ⚫ |  | ⚫ |  |  |  |  | ⚫ |  |  |  |
| Campbell 2018^26^ |  |  |  |  |  |  |  |  |  |  |  |  |  |  |  |  |  | ⚫ |  |  | ⚫ |  |  |  |  |
| Campbell 2020^27^ |  |  |  |  |  |  |  |  |  |  |  |  |  |  |  |  |  | ⚫ |  |  | ⚫ |  |  |  |  |
| Castro 2004^28^ | ⚫ | ⚫ |  |  |  |  |  |  |  |  |  |  |  |  |  |  |  |  |  |  |  |  |  | ⚫ |  |
| Chambers 2016^29^ | ⚫ |  |  |  |  |  |  |  |  |  |  |  |  |  |  |  |  |  |  |  |  | ⚫ |  |  |  |
| Chan 2013^30^ |  |  |  |  |  |  |  |  |  |  |  |  |  |  |  |  |  |  |  | ⚫ |  |  | ⚫ |  |  |
| Chan 2019^31^ |  |  |  |  |  |  |  |  |  |  |  |  |  |  |  |  |  | ⚫ | ⚫ |  | ⚫ |  |  |  |  |
| Chan 2021^32^ |  | ⚫ |  |  |  |  |  |  |  | ⚫ |  | ⚫ | ⚫ |  | ⚫ |  |  |  |  |  |  |  |  | ⚫ |  |
| Charlesworth 2013^33^ |  |  |  |  |  |  |  | ⚫ |  |  | ⚫ | ⚫ | ⚫ | ⚫ | ⚫ |  | ⚫ |  |  |  |  |  | ⚫ |  |  |
| Cocks 2013^34^ |  |  |  |  |  |  |  |  |  |  |  |  |  |  |  |  | ⚫ |  |  |  |  |  |  | ⚫ |  |
| Collins 2005^35^ | ⚫ |  |  | ⚫ |  | ⚫ |  | ⚫ |  |  |  |  |  |  |  |  |  |  |  |  |  | ⚫ |  |  |  |
| Collins 2011^36^ | ⚫ |  |  | ⚫ |  | ⚫ |  | ⚫ |  |  |  |  |  |  |  |  |  |  |  |  |  | ⚫ |  |  |  |
| Conn 2010^37^ |  |  |  |  |  |  |  |  |  | ⚫ |  | ⚫ | ⚫ | ⚫ | ⚫ | ⚫ | ⚫ |  | ⚫ | ⚫ | ⚫ |  |  |  |  |
| Connelly 2008^38^ |  |  |  |  |  |  |  |  |  |  |  |  |  |  | ⚫ | ⚫ | ⚫ | ⚫ |  | ⚫ | ⚫ |  |  |  |  |
| Cook 2014^39^ |  |  |  |  |  |  |  |  |  |  |  |  |  |  |  | ⚫ |  |  |  |  |  |  |  |  | ⚫ |
| Craig 2008^40^ | ⚫ |  |  | ⚫ |  |  |  |  |  | ⚫ | ⚫ | ⚫ | ⚫ | ⚫ | ⚫ |  | ⚫ |  |  |  |  |  |  | ⚫ |  |
| Czajkowski 2015^41^ |  |  |  |  |  | ⚫ |  | ⚫ | ⚫ |  |  |  |  |  |  | ⚫ | ⚫ | ⚫ |  | ⚫ |  | ⚫ |  |  |  |
| Damschroder 2009^42^ | ⚫ | ⚫ | ⚫ |  |  | ⚫ |  |  |  | ⚫ | ⚫ | ⚫ |  |  |  |  |  |  |  |  |  | ⚫ |  |  |  |
| Davidson 2003^43^ |  |  |  |  |  |  |  |  | ⚫ |  |  |  |  |  |  |  |  |  |  |  |  |  | ⚫ |  |  |
| De Jarlais 2004^44^ |  |  |  |  |  |  |  |  |  |  |  |  |  |  |  |  |  | ⚫ | ⚫ |  |  |  | ⚫ |  |  |
| Dixon-Woods 2004^45^ |  | ⚫ | ⚫ |  | ⚫ |  |  |  |  |  |  |  |  | ⚫ |  |  |  |  |  |  |  |  |  | ⚫ |  |
| Donald 2018^46^ |  | ⚫ |  |  |  | ⚫ |  |  | ⚫ | ⚫ |  | ⚫ | ⚫ | ⚫ | ⚫ | ⚫ | ⚫ | ⚫ | ⚫ |  |  |  |  | ⚫ |  |
| Drummond 2017^47^ |  |  |  |  |  |  |  |  |  | ⚫ | ⚫ | ⚫ |  |  | ⚫ | ⚫ | ⚫ | ⚫ |  |  | ⚫ |  |  |  |  |
| El-Kotob 2018^48^ |  |  |  |  |  |  | ⚫ | ⚫ |  | ⚫ | ⚫ | ⚫ | ⚫ | ⚫ | ⚫ |  |  |  |  |  |  |  |  | ⚫ |  |
| Eldridge 2016a ^49^ |  |  |  |  |  |  |  |  |  |  |  |  |  |  |  |  | ⚫ |  |  |  |  |  |  | ⚫ |  |
| Eldridge 2016b^50^ |  |  |  |  | ⚫ |  |  |  |  |  |  |  |  |  |  |  |  | ⚫ |  |  |  | ⚫ |  |  |  |
| Eldridge 2016c^51^ |  |  |  |  |  |  |  |  |  |  |  |  |  |  |  |  |  | ⚫ | ⚫ |  |  |  | ⚫ |  |  |
| Elfeky 2022^52^ |  |  |  |  |  |  |  |  | ⚫ |  |  |  | ⚫ |  | ⚫ |  |  |  |  |  |  |  |  |  | ⚫ |
| Evans 2013^53^ |  | ⚫ | ⚫ |  |  |  |  |  |  | ⚫ |  | ⚫ | ⚫ | ⚫ | ⚫ |  |  |  |  |  |  |  |  | ⚫ |  |
| Feeley 2009^54^ |  |  |  |  |  |  |  |  |  |  |  |  |  |  |  |  |  |  |  |  | ⚫ |  |  |  |  |
| Fletcher 2016^55^ |  |  | ⚫ |  | ⚫ |  |  |  | ⚫ |  |  |  |  | ⚫ |  | ⚫ | ⚫ |  |  |  |  | ⚫ |  |  |  |
| Freedland 2020^56^ | ⚫ |  |  |  |  | ⚫ | ⚫ | ⚫ |  |  |  |  |  |  |  | ⚫ | ⚫ |  |  |  |  |  |  | ⚫ |  |
| French 2012^57^ |  |  |  | ⚫ | ⚫ |  |  |  |  |  |  |  |  |  |  |  |  |  |  |  |  | ⚫ |  |  |  |
| Friede 2006^58^ |  |  |  |  |  |  |  |  |  |  |  |  |  |  |  |  | ⚫ |  |  |  |  |  |  |  | ⚫ |
| Friedman 2013^59^ |  |  |  |  |  |  |  |  |  |  |  |  |  |  |  |  |  | ⚫ |  | ⚫ | ⚫ |  |  |  |  |
| Gadke 2021^60^ | ⚫ | ⚫ |  |  |  |  |  |  | ⚫ | ⚫ | ⚫ | ⚫ | ⚫ | ⚫ | ⚫ |  |  |  |  | ⚫ |  |  |  | ⚫ |  |
| Giangregorio 2015^61^ |  |  |  |  |  |  |  |  |  |  |  |  |  |  |  |  |  |  |  |  |  |  |  |  |  |
| Glasgow 1999^62^ |  |  |  |  |  |  |  |  | ⚫ |  |  |  |  |  |  |  |  |  |  |  |  | ⚫ |  |  |  |
| Gonot-Schoupinsky 2019^63^ | ⚫ |  |  |  |  | ⚫ |  |  |  | ⚫ |  | ⚫ |  |  |  | ⚫ |  |  |  |  |  | ⚫ |  |  |  |
| Gooding 2018^64^ |  |  |  |  |  |  |  |  |  |  |  |  |  |  |  |  |  |  |  |  |  |  |  |  |  |
| Hallingberg 2018^65^ | ⚫ |  |  |  |  | ⚫ | ⚫ |  | ⚫ |  |  | ⚫ |  |  |  | ⚫ |  |  |  |  |  |  |  |  | ⚫ |
| Hampson 2018^66^ |  |  |  |  |  |  | ⚫ |  |  |  |  |  |  |  | ⚫ | ⚫ |  |  |  |  |  | ⚫ |  |  |  |
| Harvey 2018^67^ |  |  |  |  |  |  |  |  | ⚫ |  |  |  |  |  |  | ⚫ |  | ⚫ |  |  | ⚫ |  |  |  |  |
| Hassan 2006^68^ |  |  |  |  |  |  |  |  |  |  |  |  |  | ⚫ | ⚫ |  |  |  |  |  | ⚫ |  |  |  |  |
| Hawkins 2017^69^ |  |  | ⚫ |  |  |  |  |  |  |  |  |  |  |  |  |  |  |  |  |  |  | ⚫ |  |  |  |
| Hertzog 2008^70^ |  |  |  |  |  |  |  |  |  |  |  |  |  |  |  |  | ⚫ |  |  |  |  |  |  | ⚫ |  |
| Hoddinott 2015^71^ |  |  |  |  | ⚫ | ⚫ |  | ⚫ | ⚫ |  |  |  |  |  |  |  |  |  |  |  | ⚫ |  |  |  |  |
| Hoffmann 2014^72^ | ⚫ |  |  | ⚫ |  |  |  |  |  |  |  | ⚫ |  |  |  |  |  | ⚫ | ⚫ |  |  |  | ⚫ |  |  |
| Huang 2018^73^ |  | ⚫ | ⚫ |  | ⚫ |  |  |  |  |  | ⚫ | ⚫ |  | ⚫ | ⚫ |  |  |  |  | ⚫ |  | ⚫ |  |  |  |
| In 2017^74^ |  |  |  |  |  |  |  | ⚫ |  | ⚫ |  |  |  |  | ⚫ |  | ⚫ |  | ⚫ |  | ⚫ |  |  |  |  |
| Indig 2018^75^ |  |  |  |  |  |  |  |  |  |  |  |  |  |  |  |  |  |  |  |  |  | ⚫ |  |  |  |
| Jairath 2000^76^ |  |  |  |  |  |  |  | ⚫ |  |  |  |  |  | ⚫ |  |  | ⚫ |  |  |  | ⚫ |  |  |  |  |
| Julious 2005^77^ |  |  |  |  |  |  |  |  |  |  |  |  |  |  |  |  | ⚫ |  |  |  |  |  |  | ⚫ |  |
| Kirk 2020^78^ | ⚫ |  |  |  |  |  |  |  |  |  |  |  |  |  |  |  |  |  | ⚫ |  |  | ⚫ |  |  |  |
| Kistin 2015^79^ |  |  |  |  |  |  |  |  |  |  |  | ⚫ |  |  | ⚫ | ⚫ | ⚫ | ⚫ | ⚫ |  | ⚫ |  |  |  |  |
| Kraemer 2006^80^ |  |  |  |  |  |  |  |  |  |  |  |  |  |  |  | ⚫ | ⚫ |  |  |  | ⚫ |  |  |  |  |
| Kumar 2019^81^ |  | ⚫ | ⚫ |  |  | ⚫ |  | ⚫ | ⚫ |  | ⚫ |  |  |  |  |  |  |  |  |  |  |  |  |  | ⚫ |
| Lancaster 2004^82^ |  |  |  |  | ⚫ |  |  | ⚫ |  | ⚫ |  | ⚫ | ⚫ | ⚫ | ⚫ | ⚫ | ⚫ |  |  |  |  |  |  | ⚫ |  |
| Lancaster 2015^83^ |  |  |  |  |  |  |  |  |  |  |  |  |  |  |  | ⚫ |  |  | ⚫ |  | ⚫ |  |  |  |  |
| Lancaster 2019^84^ |  |  |  |  |  |  | ⚫ |  |  |  |  |  |  | ⚫ |  | ⚫ |  |  |  |  |  |  | ⚫ |  |  |
| Lanphear 2001^85^ |  |  |  |  |  | ⚫ |  |  |  | ⚫ | ⚫ |  |  | ⚫ |  |  |  |  |  |  | ⚫ |  |  |  |  |
| Lee 2014^86^ |  |  |  |  |  |  |  |  |  |  |  |  |  |  |  | ⚫ |  |  |  |  |  |  |  | ⚫ |  |
| Leon 2011^87^ |  |  |  |  |  |  |  | ⚫ | ⚫ | ⚫ |  |  | ⚫ | ⚫ | ⚫ |  |  |  |  |  |  |  |  | ⚫ |  |
| Levati 2016^88^ |  |  |  |  | ⚫ | ⚫ |  | ⚫ | ⚫ | ⚫ |  |  |  | ⚫ |  | ⚫ |  |  |  |  |  |  |  |  | ⚫ |
| Lewis 2021^89^ |  |  |  |  |  |  | ⚫ |  |  |  |  |  |  |  |  |  | ⚫ |  |  |  |  |  |  | ⚫ |  |
| Loudon 2015^90^ |  | ⚫ |  |  |  |  |  |  |  | ⚫ | ⚫ | ⚫ | ⚫ |  | ⚫ |  |  |  |  |  |  | ⚫ |  |  |  |
| McConnell 2018^91^ |  |  |  |  |  |  |  |  |  |  |  |  |  |  |  |  |  |  |  |  |  |  |  |  | ⚫ |
| McCrabb 2019^92^ |  |  |  |  |  |  |  |  | ⚫ |  |  |  |  |  |  |  |  |  |  |  |  |  |  |  | ⚫ |
| McCrabb 2020^93^ | ⚫ | ⚫ |  |  |  |  |  |  | ⚫ | ⚫ | ⚫ | ⚫ |  |  |  | ⚫ |  |  |  |  |  |  |  |  | ⚫ |
| McGrath 2013^94^ |  |  |  |  |  |  |  |  |  |  |  |  |  |  |  |  | ⚫ | ⚫ |  |  | ⚫ |  |  |  |  |
| Mellor 2021^95^ |  |  |  |  |  |  | ⚫ |  |  |  |  |  |  |  |  |  |  |  |  |  |  |  |  |  | ⚫ |
| Mellor 2023^96^ |  |  |  |  |  |  | ⚫ |  |  |  |  |  |  |  |  |  |  |  |  |  |  |  |  | ⚫ |  |
| Meyers 2012^97^ |  |  |  |  |  |  |  |  |  |  |  |  |  |  |  |  |  |  | ⚫ |  |  | ⚫ |  |  |  |
| Michie 2009^98^ |  |  |  | ⚫ |  |  |  |  |  |  |  |  |  |  |  |  |  | ⚫ | ⚫ |  | ⚫ |  |  |  |  |
| Milat 2011^99^ |  |  |  |  |  |  |  |  | ⚫ |  |  |  |  |  |  |  |  |  |  |  | ⚫ |  |  |  |  |
| Milat 2013^100^ |  |  |  |  |  |  |  |  | ⚫ |  |  |  |  |  |  |  |  |  |  |  |  |  |  | ⚫ |  |
| Milat 2015^101^ |  |  |  |  |  |  |  |  | ⚫ |  |  |  |  |  |  |  |  |  |  |  |  |  |  |  | ⚫ |
| Milat 2016^102^ |  |  |  |  |  |  |  |  | ⚫ |  |  |  |  |  |  |  |  |  |  |  |  |  |  | ⚫ |  |
| Miller 2021^103^ | ⚫ | ⚫ | ⚫ |  | ⚫ | ⚫ |  |  | ⚫ | ⚫ | ⚫ | ⚫ |  |  |  |  |  |  | ⚫ |  |  | ⚫ |  |  |  |
| Moffatt 2006^104^ |  |  |  |  |  |  |  |  |  |  |  |  |  | ⚫ |  |  |  |  |  |  | ⚫ |  |  |  |  |
| Mohler 2013^105^ | ⚫ | ⚫ |  |  |  | ⚫ |  |  | ⚫ | ⚫ |  | ⚫ |  |  |  |  |  |  | ⚫ |  |  |  |  |  | ⚫ |
| Mohler 2015^106^ | ⚫ | ⚫ |  |  |  | ⚫ |  |  | ⚫ | ⚫ |  | ⚫ |  |  |  |  |  |  | ⚫ |  |  |  | ⚫ |  |  |
| Moore 2011^107^ |  | ⚫ |  |  | ⚫ | ⚫ |  | ⚫ |  |  |  |  |  | ⚫ |  | ⚫ | ⚫ | ⚫ |  |  |  |  |  | ⚫ |  |
| Mummah 2016^108^ |  | ⚫ | ⚫ | ⚫ | ⚫ |  |  |  |  |  |  |  |  |  |  |  |  |  | ⚫ |  |  | ⚫ |  |  |  |
| Munir 2013^109^ |  |  | ⚫ | ⚫ | ⚫ |  |  |  | ⚫ |  |  |  |  |  |  |  |  |  |  |  |  | ⚫ |  |  |  |
| Nahum-Shani 2015^110^ | ⚫ |  |  |  |  |  |  |  |  |  |  |  |  |  |  |  |  |  | ⚫ |  |  | ⚫ |  |  |  |
| O'Brien 2014^111^ |  |  |  |  |  |  |  |  |  |  |  |  |  | ⚫ |  |  |  | ⚫ | ⚫ |  |  |  |  |  |  |
| O'Cathain 2015^112^ |  |  |  |  | ⚫ | ⚫ |  |  | ⚫ |  |  |  |  | ⚫ |  |  |  |  | ⚫ |  |  |  |  |  | ⚫ |
| O'Cathain 2019^113^ |  | ⚫ | ⚫ | ⚫ |  | ⚫ |  |  | ⚫ |  |  |  |  | ⚫ |  |  |  |  |  |  |  |  |  | ⚫ |  |
| Ogrinc 2015^114^ |  | ⚫ |  |  | ⚫ |  |  |  | ⚫ |  |  |  |  | ⚫ |  |  |  | ⚫ | ⚫ |  |  |  | ⚫ |  |  |
| O'Hara 2013^115^ | ⚫ |  | ⚫ |  |  | ⚫ |  |  | ⚫ | ⚫ |  |  |  |  |  |  |  |  | ⚫ |  | ⚫ |  |  |  |  |
| Onken 2014^116^ | ⚫ |  |  |  |  | ⚫ |  |  | ⚫ |  |  |  |  |  |  |  |  |  |  |  |  | ⚫ |  |  |  |
| Orsmond 2015^117^ |  |  |  |  |  |  |  |  |  | ⚫ | ⚫ |  |  | ⚫ | ⚫ |  |  | ⚫ |  |  |  |  |  | ⚫ |  |
| Paina 2012^118^ | ⚫ |  |  |  |  | ⚫ |  |  | ⚫ |  |  |  |  |  |  |  |  |  |  |  | ⚫ |  |  |  |  |
| Pearson 2020^119^ | ⚫ | ⚫ |  |  | ⚫ |  | ⚫ | ⚫ |  | ⚫ | ⚫ | ⚫ |  |  |  |  | ⚫ |  |  |  |  |  |  | ⚫ |  |
| Pfadenhauer 2017^120^ |  | ⚫ | ⚫ | ⚫ |  |  |  |  |  |  |  |  |  |  |  |  |  |  | ⚫ |  |  | ⚫ |  |  |  |
| Prescott 1989^121^ |  |  |  | ⚫ |  | ⚫ |  | ⚫ |  | ⚫ |  | ⚫ |  | ⚫ |  | ⚫ | ⚫ |  |  |  | ⚫ |  |  |  |  |
| Proctor 2011^122^ |  | ⚫ |  |  |  |  |  |  | ⚫ | ⚫ | ⚫ | ⚫ |  | ⚫ |  |  |  | ⚫ | ⚫ |  |  |  |  | ⚫ |  |
| Rogers 1995^123^ |  |  |  | ⚫ |  | ⚫ |  |  | ⚫ |  |  |  |  |  |  |  |  |  |  |  | ⚫ |  |  |  |  |
| Rounsaville 2001^124^ |  |  |  | ⚫ |  |  |  |  | ⚫ | ⚫ |  |  |  | ⚫ | ⚫ | ⚫ |  |  |  |  |  | ⚫ |  |  |  |
| Sandvik 1996^125^ |  |  |  |  |  |  |  |  |  |  |  |  |  |  |  |  | ⚫ |  |  |  |  |  |  | ⚫ |  |
| Shanyinde 2011^126^ |  |  |  |  |  |  |  | ⚫ |  | ⚫ | ⚫ |  | ⚫ | ⚫ | ⚫ | ⚫ | ⚫ |  |  |  |  |  |  | ⚫ |  |
| Shih 2004^127^ |  |  |  |  |  |  |  |  |  |  |  |  |  |  |  | ⚫ | ⚫ |  |  |  | ⚫ |  |  |  |  |
| Sim 2012^128^ |  |  |  |  |  |  |  |  |  |  |  |  |  |  |  |  | ⚫ |  |  |  |  |  |  | ⚫ |  |
| Sim 2019^129^ |  |  |  |  |  |  |  |  |  |  |  |  |  |  |  | ⚫ |  |  |  |  | ⚫ |  |  |  |  |
| Smith 2009^130^ |  |  | ⚫ |  | ⚫ |  |  | ⚫ |  | ⚫ |  | ⚫ | ⚫ | ⚫ | ⚫ |  | ⚫ |  |  |  |  | ⚫ |  |  |  |
| Spicer 2014^131^ |  | ⚫ | ⚫ |  |  | ⚫ |  |  | ⚫ | ⚫ | ⚫ |  |  |  |  | ⚫ |  |  |  |  |  |  |  | ⚫ |  |
| Stallard 2012^132^ |  |  |  |  |  |  |  |  |  |  |  |  |  |  |  |  | ⚫ |  |  |  |  |  |  | ⚫ |  |
| Stewart 2020^133^ |  |  |  |  |  |  |  |  |  |  |  |  |  |  | ⚫ |  |  |  | ⚫ |  |  |  |  | ⚫ |  |
| Stirman 2013^134^ | ⚫ | ⚫ |  |  |  |  |  |  |  |  |  | ⚫ |  |  |  |  |  |  |  |  |  | ⚫ |  |  |  |
| Stirman 2019^135^ | ⚫ | ⚫ |  |  |  |  |  |  |  |  |  | ⚫ |  |  |  |  |  |  |  |  |  | ⚫ |  |  |  |
| Story 2018^136^ |  |  |  |  |  |  |  |  |  |  |  |  |  |  |  | ⚫ | ⚫ | ⚫ |  |  | ⚫ |  |  |  |  |
| Subramanian 2011^137^ |  |  |  |  |  |  |  |  | ⚫ |  |  |  |  |  |  |  |  |  | ⚫ |  |  | ⚫ |  |  |  |
| Taylor 2021^138^ | ⚫ |  |  |  |  | ⚫ |  |  | ⚫ | ⚫ |  |  |  | ⚫ |  |  |  |  |  |  |  |  |  | ⚫ |  |
| Teare 2014^139^ |  |  |  |  |  |  |  | ⚫ |  |  |  |  |  |  |  | ⚫ | ⚫ |  |  |  | ⚫ |  |  |  |  |
| Teresi 2022^140^ |  |  |  |  |  |  |  | ⚫ |  | ⚫ |  | ⚫ |  | ⚫ |  | ⚫ | ⚫ |  |  |  |  |  |  | ⚫ |  |
| Thabane 2010^141^ |  |  |  |  |  |  | ⚫ | ⚫ | ⚫ | ⚫ | ⚫ | ⚫ | ⚫ | ⚫ | ⚫ | ⚫ | ⚫ | ⚫ |  |  | ⚫ |  |  |  |  |
| Thabane 2016^142^ |  |  |  |  |  |  |  |  |  |  |  |  |  |  |  |  |  | ⚫ | ⚫ |  |  |  | ⚫ |  |  |
| Thabane 2018^143^ |  |  |  |  |  |  |  | ⚫ |  |  |  |  |  |  |  |  |  |  |  |  | ⚫ |  |  |  |  |
| Thabane 2019a^144^ |  |  |  |  |  |  |  |  | ⚫ |  |  | ⚫ |  |  |  |  |  |  |  |  |  |  | ⚫ |  |  |
| Thabane 2019b^145^ |  |  |  |  |  |  |  |  |  |  |  |  |  |  |  |  |  | ⚫ |  |  | ⚫ |  |  |  |  |
| Tickle-Degnen 2013^146^ |  |  |  |  |  |  |  |  |  |  | ⚫ |  | ⚫ | ⚫ | ⚫ |  |  | ⚫ |  |  |  |  |  | ⚫ |  |
| Tong 2007^147^ |  |  |  |  |  |  |  |  |  |  |  |  |  | ⚫ |  |  |  | ⚫ | ⚫ |  |  |  | ⚫ |  |  |
| Tong 2012^148^ |  |  |  |  |  |  |  |  |  |  |  |  |  | ⚫ |  |  |  | ⚫ | ⚫ |  |  |  | ⚫ |  |  |
| van Teijlingen 2001^149^ |  |  |  |  |  |  |  | ⚫ | ⚫ |  | ⚫ | ⚫ | ⚫ | ⚫ | ⚫ |  | ⚫ |  |  |  | ⚫ |  |  |  |  |
| van Teijlingen 2002^150^ |  |  |  |  |  |  |  | ⚫ | ⚫ |  | ⚫ | ⚫ | ⚫ | ⚫ | ⚫ |  | ⚫ |  |  |  | ⚫ |  |  |  |  |
| Wandersman 2008^151^ |  |  |  |  |  |  |  |  |  |  |  |  |  |  |  |  |  |  | ⚫ |  |  | ⚫ |  |  |  |
| Watson 2007^152^ |  |  |  |  |  |  |  |  |  |  | ⚫ | ⚫ |  |  | ⚫ |  |  | ⚫ |  |  | ⚫ |  |  |  |  |
| Westlund 2017^153^ |  |  | ⚫ |  |  |  | ⚫ | ⚫ |  |  |  |  |  |  |  | ⚫ | ⚫ |  |  |  |  |  |  | ⚫ |  |
| Whitehead 2014^154^ |  |  |  |  |  |  |  |  |  |  |  |  |  |  |  |  |  | ⚫ |  |  | ⚫ |  |  |  |  |
| Whitehead 2016^155^ |  |  |  |  |  |  |  |  |  |  |  |  |  |  |  |  | ⚫ |  |  |  |  |  |  | ⚫ |  |
| Whittaker 2012^156^ |  | ⚫ | ⚫ | ⚫ |  | ⚫ |  | ⚫ | ⚫ | ⚫ |  |  |  |  |  |  |  |  |  |  |  | ⚫ |  |  |  |
| WHO 2010^157^ |  |  |  |  |  |  |  |  | ⚫ |  |  |  |  |  |  |  |  |  | ⚫ |  |  |  |  | ⚫ |  |
| WHO 2011^158^ | ⚫ | ⚫ | ⚫ |  |  |  |  |  | ⚫ | ⚫ | ⚫ | ⚫ |  | ⚫ |  |  |  |  |  |  |  |  |  | ⚫ |  |
| Wight 2015^159^ |  |  |  |  | ⚫ | ⚫ |  |  |  |  |  | ⚫ |  |  |  |  |  |  | ⚫ |  |  | ⚫ |  |  |  |
| Wilson 2015^160^ |  |  |  |  |  |  |  |  |  |  |  |  |  |  |  | ⚫ |  |  |  |  |  |  |  | ⚫ |  |
| Yamey 2011^161^ | ⚫ | ⚫ | ⚫ |  |  |  |  |  | ⚫ |  |  |  |  |  |  |  |  |  |  |  |  |  |  |  |  |
| Young 2019^162^ |  |  | ⚫ |  |  |  | ⚫ |  |  |  |  |  |  |  |  |  |  |  |  |  |  |  |  | ⚫ |  |
| **Totals** | 29 | 34 | 23 | 18 | 22 | 30 | 14 | 31 | 47 | 43 | 28 | 45 | 26 | 45 | 40 | 38 | 46 | 32 | 32 | 10 | 42 | 37 | 15 | 43 | 18 |

**References**

1. Abbott JH. The Distinction Between Randomized Clinical Trials (RCTs) and Preliminary Feasibility and Pilot Studies: What They Are and Are Not. *J Orthop Sports Phys Ther*. 2014;44(8):555-558. doi:10.2519/jospt.2014.0110

2. Albers C, Lakens D. When power analyses based on pilot data are biased: Inaccurate effect size estimators and follow-up bias. *J Exp Soc Psychol*. 2018;74:187-195. doi:10.1016/j.jesp.2017.09.004

3. Albrecht L, Archibald M, Arseneau D, Scott SD. Development of a checklist to assess the quality of reporting of knowledge translation interventions using the Workgroup for Intervention Development and Evaluation Research (WIDER) recommendations. *Implement Sci*. 2013;8(1):52. doi:10.1186/1748-5908-8-52

4. Algase DL. To Publish or Not: That Is Not the Real Pilot Study Question? *Res Theory Nurs Pract*. 2009;23(2):83-84. doi:10.1891/1541-6577.23.2.83

5. An M, Dusing SC, Harbourne RT, Sheridan SM, START-Play Consortium. What Really Works in Intervention? Using Fidelity Measures to Support Optimal Outcomes. *Phys Ther*. 2020;100(5):757-765. doi:10.1093/ptj/pzaa006

6. Arain M, Campbell MJ, Cooper CL, Lancaster GA. What is a pilot or feasibility study? A review of current practice and editorial policy. *BMC Med Res Methodol*. 2010;10(1):67. doi:10.1186/1471-2288-10-67

7. Arnold DM, Burns KEA, Adhikari NKJ, Kho ME, Meade MO, Cook DJ. The design and interpretation of pilot trials in clinical research in critical care: *Crit Care Med*. 2009;37(Supplement):S69-S74. doi:10.1097/CCM.0b013e3181920e33

8. Aschbrenner KA, Kruse G, Gallo JJ, Plano Clark VL. Applying mixed methods to pilot feasibility studies to inform intervention trials. *Pilot Feasibility Stud*. 2022;8(1):217. doi:10.1186/s40814-022-01178-x

9. Avery KNL, Williamson PR, Gamble C, et al. Informing efficient randomised controlled trials: exploration of challenges in developing progression criteria for internal pilot studies. *BMJ Open*. 2017;7(2):e013537. doi:10.1136/bmjopen-2016-013537

10. Baier RR, Jutkowitz E, Mitchell SL, McCreedy E, Mor V. Readiness assessment for pragmatic trials (RAPT): a model to assess the readiness of an intervention for testing in a pragmatic trial. *BMC Med Res Methodol*. 2019;19(1):156. doi:10.1186/s12874-019-0794-9

11. Barker PM, Reid A, Schall MW. A framework for scaling up health interventions: lessons from large-scale improvement initiatives in Africa. *Implement Sci*. 2015;11(1):12. doi:10.1186/s13012-016-0374-x

12. Barrera M, Castro FG. A Heuristic Framework for the Cultural Adaptation of Interventions. *Clin Psychol Sci Pract*. 2006;13(4):311-316. doi:10.1111/j.1468-2850.2006.00043.x

13. Barrera M, Castro FG, Strycker LA, Toobert DJ. Cultural adaptations of behavioral health interventions: A progress report. *J Consult Clin Psychol*. 2013;81(2):196-205. doi:10.1037/a0027085

14. Bartholomew LK, Parcel GS, Kok G. Intervention Mapping: A Process for Developing Theory and Evidence-Based Health Education Programs. *Health Educ Behav*. 1998;25(5):545-563. doi:10.1177/109019819802500502

15. Becker PT. Publishing pilot intervention studies. *Res Nurs Health*. 2008;31(1):1-3. doi:10.1002/nur.20268

16. Beebe LH. What Can We Learn From Pilot Studies? *Perspect Psychiatr Care*. 2007;43(4):213-218. doi:10.1111/j.1744-6163.2007.00136.x

17. Bell ML, Whitehead AL, Julious SA. Guidance for using pilot studies to inform the design of intervention trials with continuous outcomes. *Clin Epidemiol*. 2018;Volume 10:153-157. doi:10.2147/CLEP.S146397

18. Billingham SA, Whitehead AL, Julious SA. An audit of sample sizes for pilot and feasibility trials being undertaken in the United Kingdom registered in the United Kingdom Clinical Research Network database. *BMC Med Res Methodol*. 2013;13(1):104. doi:10.1186/1471-2288-13-104

19. Blatch-Jones AJ, Pek W, Kirkpatrick E, Ashton-Key M. Role of feasibility and pilot studies in randomised controlled trials: a cross-sectional study. *BMJ Open*. 2018;8(9):e022233. doi:10.1136/bmjopen-2018-022233

20. Bond C. When is a pilot not a pilot? *Int J Pharm Pract*. 2017;25(2):105-106. doi:10.1111/ijpp.12310

21. Bond C, Lancaster GA, Campbell M, et al. Pilot and feasibility studies: extending the conceptual framework. *Pilot Feasibility Stud*. 2023;9(1):24. doi:10.1186/s40814-023-01233-1

22. Borek AJ, Abraham C, Smith JR, Greaves CJ, Tarrant M. A checklist to improve reporting of group-based behaviour-change interventions. *BMC Public Health*. 2015;15(1):963. doi:10.1186/s12889-015-2300-6

23. Bowen DJ, Kreuter M, Spring B, et al. How We Design Feasibility Studies. *Am J Prev Med*. 2009;36(5):452-457. doi:10.1016/j.amepre.2009.02.002

24. Braganza MZ, Kilbourne AM. The Quality Enhancement Research Initiative (QUERI) Impact Framework: Measuring the Real-world Impact of Implementation Science. *J Gen Intern Med*. 2021;36(2):396-403. doi:10.1007/s11606-020-06143-z

25. Bugge C, Williams B, Hagen S, et al. A process for Decision-making after Pilot and feasibility Trials (ADePT): development following a feasibility study of a complex intervention for pelvic organ prolapse. *Trials*. 2013;14(1):353. doi:10.1186/1745-6215-14-353

26. Campbell MJ, Lancaster GA, Eldridge SM. A randomised controlled trial is not a pilot trial simply because it uses a surrogate endpoint. *Pilot Feasibility Stud*. 2018;4(1):130. doi:10.1186/s40814-018-0324-2

27. Campbell MJ, Mansournia MA, Lancaster G. Methods matter: pilot and feasibility studies in sports medicine. *Br J Sports Med*. 2020;54(22):1309-1310. doi:10.1136/bjsports-2020-102631

28. Castro FG, Barrera, Jr. M, Martinez, Jr. CR. The Cultural Adaptation of Prevention Interventions: Resolving Tensions Between Fidelity and Fit. *Prev Sci*. 2004;5(1):41-45. doi:10.1023/B:PREV.0000013980.12412.cd

29. Chambers DA, Norton WE. The Adaptome. *Am J Prev Med*. 2016;51(4):S124-S131. doi:10.1016/j.amepre.2016.05.011

30. Chan AW, Tetzlaff JM, Gotzsche PC, et al. SPIRIT 2013 explanation and elaboration: guidance for protocols of clinical trials. *BMJ*. 2013;346(jan08 15):e7586-e7586. doi:10.1136/bmj.e7586

31. Chan CL. A website for pilot and feasibility studies: giving your research the best chance of success. *Pilot Feasibility Stud*. 2019;5(1):122, s40814-019-0522-0526. doi:10.1186/s40814-019-0522-6

32. Chan CL, Taljaard M, Lancaster GA, Brehaut JC, Eldridge SM. Pilot and feasibility studies for pragmatic trials have unique considerations and areas of uncertainty. *J Clin Epidemiol*. 2021;138:102-114. doi:10.1016/j.jclinepi.2021.06.029

33. Charlesworth G, Burnell K, Hoe J, Orrell M, Russell I. Acceptance checklist for clinical effectiveness pilot trials: a systematic approach. *BMC Med Res Methodol*. 2013;13(1):78. doi:10.1186/1471-2288-13-78

34. Cocks K, Torgerson DJ. Sample size calculations for pilot randomized trials: a confidence interval approach. *J Clin Epidemiol*. 2013;66(2):197-201. doi:10.1016/j.jclinepi.2012.09.002

35. Collins LM, Murphy SA, Nair VN, Strecher VJ. A strategy for optimizing and evaluating behavioral interventions. *Ann Behav Med*. 2005;30(1):65-73. doi:10.1207/s15324796abm3001_8

36. Collins LM, Baker TB, Mermelstein RJ, et al. The Multiphase Optimization Strategy for Engineering Effective Tobacco Use Interventions. *Ann Behav Med*. 2011;41(2):208-226. doi:10.1007/s12160-010-9253-x

37. Conn VS, Algase DL, Rawl SM, Zerwic JJ, Wyman JF. Publishing Pilot Intervention Work. *West J Nurs Res*. 2010;32(8):994-1010. doi:10.1177/0193945910367229

38. Connelly LM. Pilot studies. *Medsurg Nurs Off J Acad Med-Surg Nurses*. 2008;17(6):411-412.

39. Cook J, Hislop J, Adewuyi T, et al. Assessing methods to specify the target difference for a randomised controlled trial: DELTA (Difference ELicitation in TriAls) review. *Health Technol Assess*. 2014;18(28). doi:10.3310/hta18280

40. Craig P, Dieppe P, Macintyre S, Michie S, Nazareth I, Petticrew M. Developing and evaluating complex interventions: the new Medical Research Council guidance. *BMJ*. Published online September 29, 2008:a1655. doi:10.1136/bmj.a1655

41. Czajkowski SM, Powell LH, Adler N, et al. From ideas to efficacy: The ORBIT model for developing behavioral treatments for chronic diseases. *Health Psychol*. 2015;34(10):971-982. doi:10.1037/hea0000161

42. Damschroder LJ, Aron DC, Keith RE, Kirsh SR, Alexander JA, Lowery JC. Fostering implementation of health services research findings into practice: a consolidated framework for advancing implementation science. *Implement Sci*. 2009;4(1):50. doi:10.1186/1748-5908-4-50

43. Davidson KW, Goldstein M, Kaplan RM, et al. Evidence-based behavioral medicine: What is it and how do we achieve it? *Ann Behav Med*. 2003;26(3):161-171. doi:10.1207/S15324796ABM2603_01

44. Des Jarlais DC, Lyles C, Crepaz N, the TREND Group. Improving the Reporting Quality of Nonrandomized Evaluations of Behavioral and Public Health Interventions: The TREND Statement. *Am J Public Health*. 2004;94(3):361-366. doi:10.2105/AJPH.94.3.361

45. Dixon-Woods M. *Integrative Approaches to Qualitative and Quantitative Evidence*. Health Development Agency; 2004.

46. Donald G. A brief summary of pilot and feasibility studies: Exploring terminology, aims, and methods. *Eur J Integr Med*. 2018;24:65-70. doi:10.1016/j.eujim.2018.10.017

47. Drummond A. Feasibility and pilot studies: Why are they important? *Br J Occup Ther*. 2017;80(6):335-336. doi:10.1177/0308022617697743

48. El-Kotob R, Giangregorio LM. Pilot and feasibility studies in exercise, physical activity, or rehabilitation research. *Pilot Feasibility Stud*. 2018;4(1):137. doi:10.1186/s40814-018-0326-0

49. Eldridge SM, Costelloe CE, Kahan BC, Lancaster GA, Kerry SM. How big should the pilot study for my cluster randomised trial be? *Stat Methods Med Res*. 2016;25(3):1039-1056. doi:10.1177/0962280215588242

50. Eldridge SM, Lancaster GA, Campbell MJ, et al. Defining Feasibility and Pilot Studies in Preparation for Randomised Controlled Trials: Development of a Conceptual Framework. Lazzeri C, ed. *PLOS ONE*. 2016;11(3):e0150205. doi:10.1371/journal.pone.0150205

51. Eldridge SM, Chan CL, Campbell MJ, et al. CONSORT 2010 statement: extension to randomised pilot and feasibility trials. *BMJ*. Published online October 24, 2016:i5239. doi:10.1136/bmj.i5239

52. Elfeky A, Treweek S, Hannes K, Bruhn H, Fraser C, Gillies K. Using qualitative methods in pilot and feasibility trials to inform recruitment and retention processes in full-scale randomised trials: a qualitative evidence synthesis. *BMJ Open*. 2022;12(4):e055521. doi:10.1136/bmjopen-2021-055521

53. Evans B, Bedson E, Bell P, et al. Involving service users in trials: developing a standard operating procedure. *Trials*. 2013;14(1):219. doi:10.1186/1745-6215-14-219

54. Feeley N, Cossette S, Côté J, Héon M, Stremler R. Meilleures pratiques en matière de recherche. 2009;41(2).

55. Fletcher A, Jamal F, Moore G, Evans RE, Murphy S, Bonell C. Realist complex intervention science: Applying realist principles across all phases of the Medical Research Council framework for developing and evaluating complex interventions. *Evaluation*. 2016;22(3):286-303. doi:10.1177/1356389016652743

56. Freedland KE. Pilot trials in health-related behavioral intervention research: Problems, solutions, and recommendations. *Health Psychol*. 2020;39(10):851-862. doi:10.1037/hea0000946

57. French SD, Green SE, O’Connor DA, et al. Developing theory-informed behaviour change interventions to implement evidence into practice: a systematic approach using the Theoretical Domains Framework. *Implement Sci*. 2012;7(1):38. doi:10.1186/1748-5908-7-38

58. Friede T, Kieser M. Sample Size Recalculation in Internal Pilot Study Designs: A Review. *Biom J*. 2006;48(4):537-555. doi:10.1002/bimj.200510238

59. Friedman L. Commentary: Why we should report results from clinical trial pilot studies. *Trials*. 2013;14(1):14. doi:10.1186/1745-6215-14-14

60. Gadke DL, Kratochwill TR, Gettinger M. Incorporating feasibility protocols in intervention research. *J Sch Psychol*. 2021;84:1-18. doi:10.1016/j.jsp.2020.11.004

61. Richards DA, Hallberg I, eds. *Complex Interventions in Health: An Overview of Research Methods*. Routledge, Taylor & Francis Group; 2015.

62. Glasgow RE, Vogt TM, Boles SM. Evaluating the public health impact of health promotion interventions: the RE-AIM framework. *Am J Public Health*. 1999;89(9):1322-1327. doi:10.2105/AJPH.89.9.1322

63. Gonot-Schoupinsky FN, Garip G. A flexible framework for planning and evaluating early-stage health interventions: FRAME-IT. *Eval Program Plann*. 2019;77:101685. doi:10.1016/j.evalprogplan.2019.101685

64. Gooding K, Phiri M, Peterson I, Parker M, Desmond N. Six dimensions of research trial acceptability: how much, what, when, in what circumstances, to whom and why? *Soc Sci Med*. 2018;213:190-198. doi:10.1016/j.socscimed.2018.07.040

65. Hallingberg B, Turley R, Segrott J, et al. Exploratory studies to decide whether and how to proceed with full-scale evaluations of public health interventions: a systematic review of guidance. *Pilot Feasibility Stud*. 2018;4(1):104. doi:10.1186/s40814-018-0290-8

66. Hampson LV, Williamson PR, Wilby MJ, Jaki T. A framework for prospectively defining progression rules for internal pilot studies monitoring recruitment. *Stat Methods Med Res*. 2018;27(12):3612-3627. doi:10.1177/0962280217708906

67. Harvey LA. Feasibility and pilot studies pave the way for definitive trials. *Spinal Cord*. 2018;56(8):723-724. doi:10.1038/s41393-018-0184-x

68. Hassan ZA, Schattner P, Mazza D. DOING A PILOT STUDY: WHY IS IT ESSENTIAL? 1(2).

69. Hawkins J, Madden K, Fletcher A, et al. Development of a framework for the co-production and prototyping of public health interventions. *BMC Public Health*. 2017;17(1):689. doi:10.1186/s12889-017-4695-8

70. Hertzog MA. Considerations in determining sample size for pilot studies. *Res Nurs Health*. 2008;31(2):180-191. doi:10.1002/nur.20247

71. Hoddinott P. A new era for intervention development studies. *Pilot Feasibility Stud*. 2015;1(1):36, s40814-015-0032-0. doi:10.1186/s40814-015-0032-0

72. Hoffmann TC, Glasziou PP, Boutron I, et al. Better reporting of interventions: template for intervention description and replication (TIDieR) checklist and guide. *BMJ*. 2014;348(mar07 3):g1687-g1687. doi:10.1136/bmj.g1687

73. Huang GD, Bull J, Johnston McKee K, Mahon E, Harper B, Roberts JN. Clinical trials recruitment planning: A proposed framework from the Clinical Trials Transformation Initiative. *Contemp Clin Trials*. 2018;66:74-79. doi:10.1016/j.cct.2018.01.003

74. In J. Introduction of a pilot study. *Korean J Anesthesiol*. 2017;70(6):601. doi:10.4097/kjae.2017.70.6.601

75. Indig D, Lee K, Grunseit A, Milat A, Bauman A. Pathways for scaling up public health interventions. *BMC Public Health*. 2018;18(1):68. doi:10.1186/s12889-017-4572-5

76. Jairath N, Hogerney M, Parsons C. The role of the pilot study: A case illustration from cardiac nursing research. *Appl Nurs Res*. 2000;13(2):92-96. doi:10.1016/S0897-1897(00)80006-3

77. Julious SA. Sample size of 12 per group rule of thumb for a pilot study. *Pharm Stat*. 2005;4(4):287-291. doi:10.1002/pst.185

78. Kirk MA, Moore JE, Wiltsey Stirman S, Birken SA. Towards a comprehensive model for understanding adaptations’ impact: the model for adaptation design and impact (MADI). *Implement Sci*. 2020;15(1):56. doi:10.1186/s13012-020-01021-y

79. Kistin C, Silverstein M. Pilot Studies: A Critical but Potentially Misused Component of Interventional Research. *JAMA*. 2015;314(15):1561. doi:10.1001/jama.2015.10962

80. Kraemer HC, Mintz J, Noda A, Tinklenberg J, Yesavage JA. Caution Regarding the Use of Pilot Studies to Guide Power Calculations for Study Proposals. *Arch Gen Psychiatry*. 2006;63(5):484. doi:10.1001/archpsyc.63.5.484

81. Kumar S, Dave P, Srivastava A, et al. Harmonizing scientific rigor with political urgency: policy learnings for identifying accelerators for scale-up from the safe childbirth checklist programme in Rajasthan, India. *BMC Health Serv Res*. 2019;19(1):273. doi:10.1186/s12913-019-4093-2

82. Lancaster GA, Dodd S, Williamson PR. Design and analysis of pilot studies: recommendations for good practice: Design and analysis of pilot studies. *J Eval Clin Pract*. 2004;10(2):307-312. doi:10.1111/j..2002.384.doc.x

83. Lancaster GA. Pilot and feasibility studies come of age! *Pilot Feasibility Stud*. 2015;1(1):1, 2055-5784-1-1. doi:10.1186/2055-5784-1-1

84. Lancaster GA, Thabane L. Guidelines for reporting non-randomised pilot and feasibility studies. *Pilot Feasibility Stud*. 2019;5(1):114, s40814-019-0499-1. doi:10.1186/s40814-019-0499-1

85. Lanphear JH. Commentary: Pilot Studies. *Educ Health Change Learn Pract*. 2001;14(1):33-35. doi:10.1080/13576280010021914

86. Lee EC, Whitehead AL, Jacques RM, Julious SA. The statistical interpretation of pilot trials: should significance thresholds be reconsidered? *BMC Med Res Methodol*. 2014;14(1):41. doi:10.1186/1471-2288-14-41

87. Leon AC, Davis LL, Kraemer HC. The role and interpretation of pilot studies in clinical research. *J Psychiatr Res*. 2011;45(5):626-629. doi:10.1016/j.jpsychires.2010.10.008

88. Levati S, Campbell P, Frost R, et al. Optimisation of complex health interventions prior to a randomised controlled trial: a scoping review of strategies used. *Pilot Feasibility Stud*. 2016;2(1):17. doi:10.1186/s40814-016-0058-y

89. Lewis M, Bromley K, Sutton CJ, McCray G, Myers HL, Lancaster GA. Determining sample size for progression criteria for pragmatic pilot RCTs: the hypothesis test strikes back! *Pilot Feasibility Stud*. 2021;7(1):40. doi:10.1186/s40814-021-00770-x

90. Loudon K, Treweek S, Sullivan F, Donnan P, Thorpe KE, Zwarenstein M. The PRECIS-2 tool: designing trials that are fit for purpose. *BMJ*. 2015;350(may08 1):h2147-h2147. doi:10.1136/bmj.h2147

91. McConnell T, Best P, Davidson G, McEneaney T, Cantrell C, Tully M. Coproduction for feasibility and pilot randomised controlled trials: learning outcomes for community partners, service users and the research team. *Res Involv Engagem*. 2018;4(1):32. doi:10.1186/s40900-018-0116-0

92. McCrabb S, Lane C, Hall A, et al. Scaling‐up evidence‐based obesity interventions: A systematic review assessing intervention adaptations and effectiveness and quantifying the scale‐up penalty. *Obes Rev*. 2019;20(7):964-982. doi:10.1111/obr.12845

93. McCrabb S, Mooney K, Elton B, Grady A, Yoong SL, Wolfenden L. How to optimise public health interventions: a scoping review of guidance from optimisation process frameworks. *BMC Public Health*. 2020;20(1):1849. doi:10.1186/s12889-020-09950-5

94. McGrath JM. Not All Studies With Small Samples Are Pilot Studies. *J Perinat Neonatal Nurs*. 2013;27(4):281-283. doi:10.1097/01.JPN.0000437186.01731.bc

95. Mellor K, Eddy S, Peckham N, et al. Progression from external pilot to definitive randomised controlled trial: a methodological review of progression criteria reporting. *BMJ Open*. 2021;11(6):e048178. doi:10.1136/bmjopen-2020-048178

96. Mellor K, Albury C, Dutton SJ, Eldridge S, Hopewell S. Recommendations for progression criteria during external randomised pilot trial design, conduct, analysis and reporting. *Pilot Feasibility Stud*. 2023;9(1):59, s40814-023-01291-01295. doi:10.1186/s40814-023-01291-5

97. Meyers DC, Durlak JA, Wandersman A. The Quality Implementation Framework: A Synthesis of Critical Steps in the Implementation Process. *Am J Community Psychol*. 2012;50(3-4):462-480. doi:10.1007/s10464-012-9522-x

98. Michie S, Fixsen D, Grimshaw JM, Eccles MP. Specifying and reporting complex behaviour change interventions: the need for a scientific method. *Implement Sci*. 2009;4(1):40, 1748-5908-4-40. doi:10.1186/1748-5908-4-40

99. Milat AJ, King L, Bauman A, Redman S. Letter – Scaling up health promotion interventions: an emerging concept in implementation science. *Health Promot J Austr*. 2011;22(3):238-238. doi:10.1071/HE11238

100. Milat AJ, King L, Bauman AE, Redman S. The concept of scalability: increasing the scale and potential adoption of health promotion interventions into policy and practice. *Health Promot Int*. 2013;28(3):285-298. doi:10.1093/heapro/dar097

101. Milat AJ, Bauman A, Redman S. Narrative review of models and success factors for scaling up public health interventions. *Implement Sci*. 2015;10(1):113. doi:10.1186/s13012-015-0301-6

102. Milat A, Newson R, King L, et al. A guide to scaling up population health interventions. *Public Health Res Pract*. 2016;26(1). doi:10.17061/phrp2611604

103. Miller CJ, Barnett ML, Baumann AA, Gutner CA, Wiltsey-Stirman S. The FRAME-IS: a framework for documenting modifications to implementation strategies in healthcare. *Implement Sci*. 2021;16(1):36. doi:10.1186/s13012-021-01105-3

104. Moffatt S, White M, Mackintosh J, Howel D. Using quantitative and qualitative data in health services research – what happens when mixed method findings conflict? [ISRCTN61522618]. *BMC Health Serv Res*. 2006;6(1):28. doi:10.1186/1472-6963-6-28

105. Möhler R, Bartoszek G, Meyer G. Quality of reporting of complex healthcare interventions and applicability of the CReDECI list - a survey of publications indexed in PubMed. *BMC Med Res Methodol*. 2013;13(1):125. doi:10.1186/1471-2288-13-125

106. Möhler R, Köpke S, Meyer G. Criteria for Reporting the Development and Evaluation of Complex Interventions in healthcare: revised guideline (CReDECI 2). *Trials*. 2015;16(1):204. doi:10.1186/s13063-015-0709-y

107. Moore CG, Carter RE, Nietert PJ, Stewart PW. Recommendations for Planning Pilot Studies in Clinical and Translational Research. *Clin Transl Sci*. 2011;4(5):332-337. doi:10.1111/j.1752-8062.2011.00347.x

108. Mummah SA, Robinson TN, King AC, Gardner CD, Sutton S. IDEAS (Integrate, Design, Assess, and Share): A Framework and Toolkit of Strategies for the Development of More Effective Digital Interventions to Change Health Behavior. *J Med Internet Res*. 2016;18(12):e317. doi:10.2196/jmir.5927

109. Munir F, Kalawsky K, Wallis DJ, Donaldson-Feilder E. Using intervention mapping to develop a work-related guidance tool for those affected by cancer. *BMC Public Health*. 2013;13(1):6. doi:10.1186/1471-2458-13-6

110. Nahum-Shani I, Hekler EB, Spruijt-Metz D. Building health behavior models to guide the development of just-in-time adaptive interventions: A pragmatic framework. *Health Psychol*. 2015;34(Suppl):1209-1219. doi:10.1037/hea0000306

111. O’Brien BC, Harris IB, Beckman TJ, Reed DA, Cook DA. Standards for Reporting Qualitative Research: A Synthesis of Recommendations. *Acad Med*. 2014;89(9):1245-1251. doi:10.1097/ACM.0000000000000388

112. O’Cathain A, Hoddinott P, Lewin S, et al. Maximising the impact of qualitative research in feasibility studies for randomised controlled trials: guidance for researchers. *Pilot Feasibility Stud*. 2015;1(1):32. doi:10.1186/s40814-015-0026-y

113. O’Cathain A, Croot L, Duncan E, et al. Guidance on how to develop complex interventions to improve health and healthcare. *BMJ Open*. 2019;9(8):e029954. doi:10.1136/bmjopen-2019-029954

114. Ogrinc G, Davies L, Goodman D, Batalden P, Davidoff F, Stevens D. Standards for QUality Improvement Reporting Excellence 2.0: revised publication guidelines from a detailed consensus process. *J Surg Res*. 2016;200(2):676-682. doi:10.1016/j.jss.2015.09.015

115. O’Hara BJ, Phongsavan P, King L, et al. “Translational formative evaluation”: critical in up-scaling public health programmes. *Health Promot Int*. 2014;29(1):38-46. doi:10.1093/heapro/dat025

116. Onken LS, Carroll KM, Shoham V, Cuthbert BN, Riddle M. Reenvisioning Clinical Science: Unifying the Discipline to Improve the Public Health. *Clin Psychol Sci*. 2014;2(1):22-34. doi:10.1177/2167702613497932

117. Orsmond GI, Cohn ES. The Distinctive Features of a Feasibility Study: Objectives and Guiding Questions. *OTJR Occup Particip Health*. 2015;35(3):169-177. doi:10.1177/1539449215578649

118. Paina L, Peters DH. Understanding pathways for scaling up health services through the lens of complex adaptive systems. *Health Policy Plan*. 2012;27(5):365-373. doi:10.1093/heapol/czr054

119. Pearson N, Naylor PJ, Ashe MC, Fernandez M, Yoong SL, Wolfenden L. Guidance for conducting feasibility and pilot studies for implementation trials. *Pilot Feasibility Stud*. 2020;6(1):167. doi:10.1186/s40814-020-00634-w

120. Pfadenhauer LM, Gerhardus A, Mozygemba K, et al. Making sense of complexity in context and implementation: the Context and Implementation of Complex Interventions (CICI) framework. *Implement Sci*. 2017;12(1):21. doi:10.1186/s13012-017-0552-5

121. Prescott PA, Soeken KL. The Potential Uses of Pilot Work: *Nurs Res*. 1989;38(1):60. doi:10.1097/00006199-198901000-00015

122. Proctor E, Silmere H, Raghavan R, et al. Outcomes for Implementation Research: Conceptual Distinctions, Measurement Challenges, and Research Agenda. *Adm Policy Ment Health Ment Health Serv Res*. 2011;38(2):65-76. doi:10.1007/s10488-010-0319-7

123. Rogers EM. *Diffusion of Innovations*. 4th ed. Free Press; 1995.

124. Rounsaville BJ, Carroll KM, Onken LS. A stage model of behavioral therapies research: Getting started and moving on from stage I. *Clin Psychol Sci Pract*. 2001;8(2):133-142. doi:10.1093/clipsy.8.2.133

125. Sandvik L, Erikssen J, Mowinckel P, Rødland EA. A METHOD FOR DETERMINING THE SIZE OF INTERNAL PILOT STUDIES. *Stat Med*. 1996;15(14):1587-1590. doi:10.1002/(SICI)1097-0258(19960730)15:14<1587::AID-SIM279>3.0.CO;2-F

126. Shanyinde M, Pickering RM, Weatherall M. Questions asked and answered in pilot and feasibility randomized controlled trials. *BMC Med Res Methodol*. 2011;11(1):117. doi:10.1186/1471-2288-11-117

127. Shih WJ, Ohman-Strickland PA, Lin Y. Analysis of pilot and early phase studies with small sample sizes. *Stat Med*. 2004;23(12):1827-1842. doi:10.1002/sim.1807

128. Sim J, Lewis M. The size of a pilot study for a clinical trial should be calculated in relation to considerations of precision and efficiency. *J Clin Epidemiol*. 2012;65(3):301-308. doi:10.1016/j.jclinepi.2011.07.011

129. Sim J. Should treatment effects be estimated in pilot and feasibility studies? *Pilot Feasibility Stud*. 2019;5(1):107. doi:10.1186/s40814-019-0493-7

130. Smith LJ, Harrison MB. Framework for Planning and Conducting Pilot Studies. Published online 2009.

131. Spicer N, Bhattacharya D, Dimka R, et al. ‘Scaling-up is a craft not a science’: Catalysing scale-up of health innovations in Ethiopia, India and Nigeria. *Soc Sci Med*. 2014;121:30-38. doi:10.1016/j.socscimed.2014.09.046

132. Stallard N. Optimal sample sizes for phase II clinical trials and pilot studies. *Stat Med*. 2012;31(11-12):1031-1042. doi:10.1002/sim.4357

133. Stewart AL, Nápoles AM, Piawah S, Santoyo-Olsson J, Teresi JA. Guidelines for Evaluating the Feasibility of Recruitment in Pilot Studies of Diverse Populations: An Overlooked but Important Component. *Ethn Dis*. 2020;30(Suppl):745-754. doi:10.18865/ed.30.S2.745

134. Stirman SW, Miller CJ, Toder K, Calloway A. Development of a framework and coding system for modifications and adaptations of evidence-based interventions. *Implement Sci*. 2013;8(1):65. doi:10.1186/1748-5908-8-65

135. Wiltsey Stirman S, Baumann AA, Miller CJ. The FRAME: an expanded framework for reporting adaptations and modifications to evidence-based interventions. *Implement Sci*. 2019;14(1):58. doi:10.1186/s13012-019-0898-y

136. Story DA, Leslie K, French C. Feasibility and Pilot Studies: Small Steps before Giant Leaps. *Anaesth Intensive Care*. 2018;46(1):11-12. doi:10.1177/0310057X1804600103

137. Subramanian S, Naimoli J, Matsubayashi T, Peters DH. Do we have the right models for scaling up health services to achieve the Millennium Development Goals? *BMC Health Serv Res*. 2011;11(1):336. doi:10.1186/1472-6963-11-336

138. Taylor SP, Kowalkowski MA. Using Implementation Science-Guided Pilot Studies to Assess and Improve the Informativeness of Clinical Trials. *J Gen Intern Med*. 2021;36(2):533-536. doi:10.1007/s11606-020-06220-3

139. Teare MD, Dimairo M, Shephard N, Hayman A, Whitehead A, Walters SJ. Sample size requirements to estimate key design parameters from external pilot randomised controlled trials: a simulation study. *Trials*. 2014;15(1):264. doi:10.1186/1745-6215-15-264

140. Teresi JA, Yu X, Stewart AL, Hays RD. Guidelines for Designing and Evaluating Feasibility Pilot Studies. *Med Care*. 2022;60(1):95-103. doi:10.1097/MLR.0000000000001664

141. Thabane L, Ma J, Chu R, et al. A tutorial on pilot studies: the what, why and how. *BMC Med Res Methodol*. 2010;10(1):1. doi:10.1186/1471-2288-10-1

142. Thabane L, Hopewell S, Lancaster GA, et al. Methods and processes for development of a CONSORT extension for reporting pilot randomized controlled trials. *Pilot Feasibility Stud*. 2016;2(1):25. doi:10.1186/s40814-016-0065-z

143. Thabane L, Lancaster G. Improving the efficiency of trials using innovative pilot designs: the next phase in the conduct and reporting of pilot and feasibility studies. *Pilot Feasibility Stud*. 2018;4(1):14, s40814-017-0159-2. doi:10.1186/s40814-017-0159-2

144. Discussion Panel, Thabane L, Cambon L, et al. Population health intervention research: what is the place for pilot studies? *Trials*. 2019;20(1):309. doi:10.1186/s13063-019-3422-4

145. Thabane L, Lancaster G. A guide to the reporting of protocols of pilot and feasibility trials. *Pilot Feasibility Stud*. 2019;5(1):37, s40814-019-0423-0428. doi:10.1186/s40814-019-0423-8

146. Tickle-Degnen L. Nuts and Bolts of Conducting Feasibility Studies. *Am J Occup Ther*. 2013;67(2):171-176. doi:10.5014/ajot.2013.006270

147. Tong A, Sainsbury P, Craig J. Consolidated criteria for reporting qualitative research (COREQ): a 32-item checklist for interviews and focus groups. *Int J Qual Health Care*. 2007;19(6):349-357. doi:10.1093/intqhc/mzm042

148. Tong A, Flemming K, McInnes E, Oliver S, Craig J. Enhancing transparency in reporting the synthesis of qualitative research: ENTREQ. *BMC Med Res Methodol*. 2012;12(1):181. doi:10.1186/1471-2288-12-181

149. Van Teijlingen ER, Rennie AM, Hundley V, Graham W. The importance of conducting and reporting pilot studies: the example of the Scottish Births Survey. *J Adv Nurs*. 2001;34(3):289-295. doi:10.1046/j.1365-2648.2001.01757.x

150. Van Teijlingen E, Hundley V. The importance of pilot studies. *Nurs Stand*. 2002;16(40):33-36. doi:10.7748/ns2002.06.16.40.33.c3214

151. Wandersman A, Duffy J, Flaspohler P, et al. Bridging the Gap Between Prevention Research and Practice: The Interactive Systems Framework for Dissemination and Implementation. *Am J Community Psychol*. 2008;41(3-4):171-181. doi:10.1007/s10464-008-9174-z

152. Watson R, Atkinson I, Rose K. Editorial: Pilot studies: to publish or not? *J Clin Nurs*. 2007;16(4):619-620. doi:10.1111/j.1365-2702.2006.01830.x

153. Westlund E, Stuart EA. The Nonuse, Misuse, and Proper Use of Pilot Studies in Experimental Evaluation Research. *Am J Eval*. 2017;38(2):246-261. doi:10.1177/1098214016651489

154. Whitehead AL, Sully BGO, Campbell MJ. Pilot and feasibility studies: Is there a difference from each other and from a randomised controlled trial? *Contemp Clin Trials*. 2014;38(1):130-133. doi:10.1016/j.cct.2014.04.001

155. Whitehead AL, Julious SA, Cooper CL, Campbell MJ. Estimating the sample size for a pilot randomised trial to minimise the overall trial sample size for the external pilot and main trial for a continuous outcome variable. *Stat Methods Med Res*. 2016;25(3):1057-1073. doi:10.1177/0962280215588241

156. Whittaker R, Merry S, Dorey E, Maddison R. A Development and Evaluation Process for mHealth Interventions: Examples From New Zealand. *J Health Commun*. 2012;17(sup1):11-21. doi:10.1080/10810730.2011.649103

157. World Health Organization, ExpandNet. Nine steps for developing a scaling-up strategy. *Neuf Étapes Pour Élabor Une Strat Passage À Gd Léchelle*. Published online 2010. Accessed July 13, 2023. https://apps.who.int/iris/handle/10665/44432

158. World Health Organization, ExpandNet. Beginning with the end in mind: planning pilot projects and other programmatic research for successful scaling up. *Avoir À L’esprit Dès Début Planif Proj Pilot D’autres Rech Program Pour Un Passage À Gd Échelle Réussi*. Published online 2011. Accessed July 13, 2023. https://apps.who.int/iris/handle/10665/44708

159. Wight D, Wimbush E, Jepson R, Doi L. Six steps in quality intervention development (6SQuID). *J Epidemiol Community Health*. 2016;70(5):520-525. doi:10.1136/jech-2015-205952

160. Wilson DT, Walwyn REA, Brown J, Farrin AJ, Brown SR. Statistical challenges in assessing potential efficacy of complex interventions in pilot or feasibility studies. *Trials*. 2015;16(S2):O90. doi:10.1186/1745-6215-16-S2-O90

161. Yamey G. Scaling Up Global Health Interventions: A Proposed Framework for Success. *PLoS Med*. 2011;8(6):e1001049. doi:10.1371/journal.pmed.1001049

162. Young H, Goodliffe S, Madhani M, et al. Co-producing Progression Criteria for Feasibility Studies: A Partnership between Patient Contributors, Clinicians and Researchers. *Int J Environ Res Public Health*. 2019;16(19):3756. doi:10.3390/ijerph16193756
