# Supplementary material for: Consolidated guidance for behavioral intervention pilot and feasibility studies
Source: Pilot Feasibility Stud. 2024 Apr 6;10:57. doi: 10.1186/s40814-024-01485-5 (PMC10998328; doi:10.1186/s40814-024-01485-5)
Supplement: Supplementary file 2 — Additional file 2. Summary table of considerations. [file 40814_2024_1485_MOESM2_ESM.docx]

| **Theme** | **Subtheme** | **Definition** | **Considerations** |
| --- | --- | --- | --- |
| Intervention Design | Adaptations and Tailoring | Adaptations and tailoring refer to any deliberate changes to the design or delivery of an intervention, with the goal of improving fit or effectiveness in a given context (Stirman et al., 2019). | •Where components of the intervention are adapted/tailored, details of who was involved (e.g., investigative team, key stakeholders, participants) in the decisions (see 1.3. Stakeholder Engagement and Co-Production), when the adaptations/tailoring occurred, and how and why the modification(s) were made need to be clearly reported.  •How the proposed adaptations/tailoring address the issues/challenges observed in the intervention need to be clearly reported along with justification for why these changes should result in an improved design.  •Whether the adaptations/tailoring occurred a priori or during the conduct of the study should be clearly described.  •The intervention component of PFS can be conducted in a rigorous fashion yet be flexible enough to allow for minor adaptations or tailoring (in composition, format, design, etc.) when justified and in response to emerging feasibility indicators.  •If substantial adaptations are made to the intervention, such that the adaptations may influence feasibility indicators or behavioral outcomes, re-testing of the PFS prior to progression is justifiable (see 2.1. Iteration and Intervention Refinement). Adaptations/tailoring occurring under these circumstances should refer to any a priori progression criteria specifications (see 2.2. Progression Criteria). |
|  | Site Selection and Context | Site selection refers to the location in which a pilot and/or feasibility study will be delivered. Context refers to the factors that form the setting of the intervention, including location, culture, environment, and situation (McKay et al., 2019; Pearson et al., 2020). | •Whenever feasible, researchers should choose sites for PFS that are representative of those anticipated in the future larger-scale trial.  •Purposeful selection of sites can be used to ensure an intervention is tested in an appropriate range of contexts.  •A rationale for the sites selected should be clearly stated along with how the sites and context reflect what is anticipated in the future larger-scale trial.  •Key characteristics of the sites and context should be reported.  •The context of intervention delivery and any information that suggests contextual elements may impact the feasibility or future efficacy of the intervention should be clearly reported.  •Where context is known or hypothesized to influence the implementation and/or feasibility of an intervention, including more than one site may be necessary. |
|  | Stakeholder Engagement and Co-Production | Stakeholder engagement and co-production refers to the use of partnerships with individuals, communities, and service providers to aid in the development and implementation of an intervention.  (Hawkins et al., 2017) | •PFS should be, whenever possible, co-designed/co-created or informed by key stakeholder (e.g., community and professional) perspectives throughout all stages of design and implementation.  •Whenever possible, pro-equity approaches that ensure the unique considerations and perspectives around an intervention's acceptability, safety, etc. and participation in and ownership of research from minority and vulnerable populations, should be used.  •The processes by which the PFS was co-designed, including who was consulted, why, when they were consulted, and how their input was obtained, should be clearly described. |
|  | Theory Usage | Theory usage refers to the utilization of any conceptual or theoretical model to inform aspects of the pilot and/or feasibility study that are mechanisms of change (O’Cathain et al., 2019). | • Researchers, where relevant, should include details about one or more theories of change (e.g., intervention activities, mechanisms) which informed aspects of the pilot and/or feasibility study, including whether components of the intervention are theoretically or practically informed. |
|  | Well Defined Problem and Aims | Well-defined problems and aims refers to the focused research questions/objectives used to guide the design, conduct, and analyses of pilot and/or feasibility studies (O’Cathain et al., 2019). | • Pilot and/or feasibility studies should be guided by clear and focused research questions related primarily to the feasibility of the intervention and prospects of subsequent scale-up to a larger-scale trial. These well formulated research questions should be answered by appropriate and transparent methodology that uses both quantitative and qualitative data.  • Where appropriate, the pilot and/or feasibility study proposal and report should define a clinically important or public health problem for which researchers are designing, refining, or adapting an intervention. |
| Study Design | Iteration and Intervention Refinement | Iteration and intervention refinement refers to the re-testing of a pilot and/or feasibility study to further refine intervention components before scaling to a larger trial (McCrabb et al., 2020). | • If the conclusion of the pilot and/or feasibility study is to make significant adjustments to either the study design or the intervention, then it should be acknowledged that the pilot and/or feasibility data are not sufficient to justify proceeding further and a second pilot and/or feasibility study is necessary to establish feasibility before testing the intervention in a larger-scale, well-powered trial. Any potential changes (adaptations/tailoring) should be clearly documented and information about how and why the changes are to be made stated (see 1.1. Adaptations and Tailoring).  • The decision to conduct multiple iterations of a pilot and/or feasibility study can be pragmatic or theoretical and based on factors including the perceived confidence the redesign will sufficiently address the identified problems.  • Conclusions from a pilot and/or feasibility study should include whether the intervention, in its current form, is ready for a future trial or if modifications are needed (and if so, details of them), and whether they are substantial enough to warrant another pilot and/or feasibility study. |
|  | Progression Criteria | Progression criteria are a set of a priori benchmarks or thresholds regarding key feasibility markers that inform decisions about whether to proceed, to proceed with changes, or not to proceed from the pilot and/or feasibility study to a future study, either a main trial or another pilot and/or feasibility study (Eldridge et al., 2021). | • Pilot and/or feasibility studies should include a set of progression criteria which are used to inform decisions about whether to proceed, proceed with changes, or not to proceed to a larger-scale study.  • Progression criteria should be determined a priori and be based on either evidence from previously published/conducted research, or a sound rationale provided.  • Decisions whether to proceed should also be informed by contextual, temporal and partnership factors that evolve over the course of the pilot and/or feasibility.  • Progression criteria should be made for feasibility metrics such as recruitment rate, retention/drop-out rate, acceptability, implementation/fidelity, and other appropriate feasibility indicators where appropriate.  • Progression decisions can also include evidence of potential impact (see 5.2. Preliminary Impact)  • Qualitative data may also provide useful information about possible changes to the pilot and/or feasibility study if progression criteria have not been met.  • Progression criteria decisions can be in the form of a “Go/No Go” system or a “Stop Light” (red/amber/green) system, indicating no progression, progression with changes, or progression with no changes.  • Deviations from the application of progression criteria may be justified if researchers are confident that a proposed solution will address the problem at a larger scale and can provide strong theoretical and/or empirical evidence to support their assertion (see 1.1. Adaptations/Tailoring). |
|  | Randomization and Control Groups | Randomization refers to the process of using random chance to allocate units (individuals or settings/clusters) to one or more interventions. Randomization can be used to separate units into distinct groups or randomization within a unit for when and what intervention(s) they may receive (order and timing). A control/comparator condition can take two primary forms and serves as the counterfactual. A control/comparator group is a group of participants (and/or settings/clusters) allocated to receive differing amounts or types of intervention(s) being tested. A baseline period can serve as a control/comparator condition for studies employing single arm or individual level interventions (e.g., N-of-1, micro-randomization).  (D’Agostino & Kwan, 1995) | • Not every pilot/feasibility study needs to include two or more groups nor employ random allocation.  • The presence of a control/comparator group or randomization can be included if it reflects the aims and objectives of the study.  • Control groups can take numerous forms and should be reflective of the objectives of the study, the context within which the intervention is tested, and acceptability by the target population.  • When randomization is employed, methods of randomization should be clearly described to ensure reproducibility.  • If a control/comparator group is present, feasibility indicators collected on the intervention group should also be collected on the control group where appropriate (e.g., feasibility of data collection, acceptability of randomization). |
|  | Scale-Up | Scale-up refers to the process of delivering and evaluating an intervention in progressively larger studies, beginning with testing an intervention within one or more pilot and/or feasibility studies and moving towards larger studies of the same, or similar, intervention. It is a “deliberate effort to increase the impact of successfully tested health intervention so as to benefit more people and foster policy and program development on a lasting basis” (WHO, 2010). | • Pilot and/or feasibility studies should be developed with the intent for future testing in large-scale trials and beyond.  • Researchers should consider plans for later-phase research on the intervention and explain how information gathered from the pilot and/or feasibility study will be used to answer key questions surrounding uncertainty of the intervention or the design or conduct of a progressively larger future study.  • Issues regarding adoption, implementation, and maintenance of the intervention over progressively larger studies can be considered at both the design and conduct phases of the pilot and/or feasibility study.  • Efforts should be made to ensure key features of the pilot and/or feasibility trial be similar to those in the future large-scale trial. These include the amount of support to implement the intervention, characteristics of who delivers the intervention, the target population, the duration under which the intervention is tested, and the measures employed.  • Where differences are anticipated between pilot and/or feasibility testing and the larger-scale trial, a complete description of these differences should be provided along with a clear justification of how the changes may or may not impact the intervention. |
| Conduct of Trial | Measurement and Data Collection | Measurement and data collection refers to any tools, devices, instruments, personnel, and time required to assess feasibility or outcomes related to an intervention. | • Pilot and/or feasibility studies can assess the feasibility and appropriateness of measurement and data collection procedures including:   - how/if the data can be collected - the acceptability of the measurements and data collection procedures (e.g., burden) - if the measures are valid for the outcomes in question   • Where applicable, measurements and data collection procedures should closely resemble those anticipated for the well-powered trial.  • The reporting of measurement and data collection procedures should be sufficiently detailed to permit standardized data collection, including information about why the measurements were selected and how they were administered, scored, and interpreted.  • Information about the feasibility and appropriateness of measurement and data collection procedures can consist of both quantitative and qualitative data sources. |
|  | Recruitment | Recruitment refers to the procedures used to identify and select potential participants (individuals and/or settings/clusters) and enroll them into a study. Recruitment rate is the proportion of eligible participants or settings/clusters who are enrolled at the baseline of an intervention compared to the eligible target population (Hallingberg et al, 2018). | • Recruitment procedures should be clearly described, with any strategies designed to maximize recruitment fully detailed.  • Information should include details of procedures used to recruit at the individual and setting/cluster levels, where appropriate.  • Recruitment information should include the following, where appropriate:   - Proportion of eligible units (e.g., individuals, settings) recruited - The start and end dates of the recruitment periods - Number of participants recruited per setting/cluster, overall, and number of settings/clusters - Number of potential participants screened, eligible, consented, and enrolled in study - Reasons for non-recruitment/non-consent - Acceptability of recruitment strategies   • Details should be provided about the recruitment processes used, measures of their success, what worked, and what may need to be altered for future studies. |
|  | Retention | Retention (attrition/drop-out) is the proportion of enrolled participants who are present throughout the full length of the intervention (Hallingberg et al, 2018). | • Researchers conducting pilot and/or feasibility studies should ensure retention rates are measured.  • Where possible, assessments can be made to identify differences in retention across groups.  • Reasons why individuals leave a study can be collected and analyzed to investigate whether particular factors are associated with retention.  • Procedures should clearly describe protocols used to assist with retaining participants during delivery of the intervention and any post-intervention follow-up time periods, where appropriate.  • Retention-related information can include both quantitative and qualitative data sources. |
| Implementation of Intervention | Acceptability | Acceptability is a perception/notion that an intervention or various aspects of an intervention are favorable, agreeable, palatable, enjoyable, satisfactory, valued, appropriate, and/or have a wider fit within a system. It relates to how users “feel” about an intervention. (Gooding et al., 2018). | • Researchers should clearly define what is meant by “acceptability” for a given study, at what levels (e.g., individual, deliverer, setting) it will be assessed, and by what methods (e.g., surveys, interviews). This should be based upon the nature of the intervention and its constituent components, target population, setting level characteristics, and key stakeholders.  • Measures of acceptability can be pre-defined and included in both the pilot/feasibility studies and large-scale trial stages.  • Acceptability should be captured, at minimum, from the end user (intervention participants). Acceptability can also be captured from those involved with delivering the intervention, along with anyone else involved in the implementation process.  • Acceptability, as defined for a given study, can be assessed for participants in control conditions where appropriate (e.g., acceptability of randomization to active comparator, acceptability of data collection procedures).  • Researchers can use both quantitative (e.g., surveys) and qualitative (e.g., interviews) methods to assess acceptability. |
|  | Fidelity | Fidelity is the degree to which an intervention is delivered as intended and the quality of that delivery. (Mihee et al., 2020; Durlak & Dupree, YEAR). | • Researchers should clearly define what is meant by “fidelity” for a given study, at what levels (e.g., individual, deliverer, setting) it will be assessed, and by what methods (e.g., surveys, interviews).  • Measures of fidelity should be pre-defined with all intervention components listed.  • Fidelity can consist of information about how an intervention will be delivered, for whom, what the intervention consists of, and when and where (context) the intervention will be delivered.  • If strategies are used to encourage fidelity (e.g., a manualized intervention, feedback to those delivering the intervention) these should be reported.  • Factors influencing fidelity can be assessed and, where appropriate, linked to feasibility outcomes. |
|  | Cost and Resources | Costs and resources refer to the investments and assets required to develop, implement, and sustain an intervention (Proctor et al., 2011; Pearson et al., 2020). | • Pilot and/or feasibility studies can include assessments of the costs and required resources of conducting an intervention.  • In some pilot/feasibility studies costs and resources may include the following:   - Monetary costs associated with training, supervision, and recruitment of both stakeholders and participants, incentivization, facilities, materials, and intervention component development and delivery. - Opportunity costs/time demands associated with completing the intervention by participants and delivering the intervention by providers.   • Researchers can collect information to determine the feasibility of measuring the costs associated with the intervention, with this information used to inform a more well-defined cost analysis/economic evaluation in a larger-scale trial.  • Researchers should keep in mind that some costs associated with the intervention will be fixed (one-time costs) and some will be recurring during the successful scale-up and sustainment of the intervention. |
| Statistical Analysis | Sample Size | Sample size refers to the number of participants (or groups/clusters) in a given study (Teare et al., 2014). | • The sample size of a pilot and/or feasibility study should be based on the feasibility objectives of the study.  • Sample sizes do not have to be based upon a formal sample size calculation (i.e., power).  • Sample sizes should be pre-specified and justified.  • Sample size estimates should consider representativeness of the target population or subgroup, setting, and other relevant contextual aspects that may influence how and why an intervention works.  • Sample characteristics should be clearly described and may refer to individuals and/or clusters (e.g., churches, workplaces, neighborhoods, schools).  • Where relevant, studies should clearly report factors impacting the sample size (e.g., availability of funds, time constraints).  • Investigators are encouraged to report the a priori power achieved of the sample size selected for a pilot/feasibility study. |
|  | Preliminary Impact | Preliminary impact is the ability of an intervention, during a pilot and/or feasibility study to produce a desired or intended result. (Leon et al., 2011). | • Pilot and/or feasibility studies need not be powered to detect statistically significant differences in outcomes, but one or more outcomes, as appropriate to the research, can be assessed.  • When outcomes are collected, changes in outcome data can be used to aid in decisions regarding the conduct of a subsequent larger-scale trial.  • In many cases, it may be necessary to demonstrate an intervention “moves” outcomes in the appropriate direction and are not causing harm. In this scenario, it is recommended statistical testing can be performed but to avoid the interpretation of p-values as conclusive evidence of an intervention’s impact in a larger-scale trial.  • Interpretations of performed statistical tests should not include a justification for (or against) proceeding to a subsequent large-scale intervention or for making claims about the likely success of the study. Interpretations should help guide, but not dominate, the decision to proceed to a large-scale intervention.  • Investigators should avoid using language such as “statistically significant” to describe their interpretation of outcomes from a pilot/feasibility study.  • Where pilot and/or feasibility estimates of impact on primary, secondary, or tertiary outcomes are reported these should be pre-specified, with point estimates and a measure of variability reported for all time points.  • For studies presenting both feasibility and outcome data, outcome data should be relegated to a secondary or exploratory focus. |
| Reporting | Pre-Registration and Protocol Publishing | Pre-registration and protocol publishing refers to an a priori process of documenting planned intervention design and analyses (APA, 2022). | • Pre-registration and a protocol made publicly available (via peer-reviewed journal, pre-print server, or other forms of public dissemination) contributes to transparency and ensures that changes between what is planned, what is conducted, and what is ultimately reported are communicated and justified. |
|  | Study Labeling | Study labeling refers to naming/presenting a pilot and/or feasibility study with appropriate naming conventions for the study being conducted. | • At minimum, researchers should make sure studies are clearly labeled to indicate their preliminary nature and reflect the aims and objectives of the study in both the title and abstract with either “pilot”, “feasibility”, “proof-of-concept”, or other relevant label(s). |
|  | Framework and Guideline Usage | The utilization of published frameworks/guidelines to guide the development, implementation, and reporting of pilot and/or feasibility studies (Pfledderer et al., 2022). | • Where possible, researchers should choose an appropriate framework to structure pilot and/or feasibility studies and use it to guide the design, conduct, analysis, and reporting of said study.  • Findings from pilot and/or feasibility studies should be disseminated in a way that adheres to reporting guidelines to facilitate transparency and allow for replication. |
